# MiR-125a-5p in extracellular vesicles of neural stem cells acts as a crosstalk signal modulating neuroinflammatory microenvironment to alleviate cerebral ischemia-reperfusion injury

**DOI:** 10.7150/thno.115993

**Published:** 2025-06-12

**Authors:** Qingyue Liu, Heran Ma, Jing Liao, Zihan Zhu, Hongyuan Chen, Dong Sun, Longkun Wang, Lu Lu, Xiaowei Chen, Xinke Zhang, Fengshan Wang

**Affiliations:** 1 Key Laboratory of Chemical Biology (Ministry of Education), Institute of Biochemical and Biotechnological Drug, School of Pharmaceutical Sciences, Shandong University, Jinan 250012, China.; 2 Qilu Cell Therapy Technology Co., Ltd, Jinan 250000, China.; 3 Department of General Surgery, Shandong Provincial Hospital Affilated to Shandong First Medical University, Jinan 250021, China.; 4 Department of Gastrointestinal Surgery, Shandong Cancer Hospital and Institute, Shandong First Medical University and Shandong Academy of Medical Science, Jinan 250012, China.; 5 Department of Pharmacy, Tianjin Anding Hospital, Tianjin 300000, China.; 6 Department of Pharmacy, Peking Union Medical College Hospital, Beijing 100010, China.; 7 Beijing Yinfeng Dingcheng Biological Engineering Technology Limited Liability Company, Beijing 100176, China.

**Keywords:** cerebral ischemia-reperfusion injury, extracellular vesicles, miR-125a-5p, *IKBKG*, neuroinflammation

## Abstract

**Rationale:** Ischemic stroke is the second leading cause of death worldwide. Ischemia-reperfusion injury plays a major role in brain function damage and leads to disability. Currently, there are no ideal therapeutic methods for preventing and treating ischemia-reperfusion injury. Extracellular vesicles (EVs) are a promising therapy for cerebral ischemia-reperfusion injury (CIRI). The main purpose of this study was to identify the pivotal miRNAs in EVs that affect functional recovery following CIRI, develop engineered EVs encapsulating key miRNAs, and identify the underlying mechanisms.

**Methods:** Next-generation sequencing was used to identify the crucial differentiating ingredients between EVs from normoxia- and hypoxia-conditioned human neural stem cells (hNSCs). HNSC EVs were electroporated with miR-125a-5p mimics and characterized using nanoparticle tracking analysis and electron microscopy. The role and mechanism by which EV-packaged miR-125a-5p mediates CIRI were investigated *in vitro* and *in vivo*.

**Results:** In the present study, miR-125a-5p derived from the EVs of hNSCs was found to signal the crosstalk between different cells, such as microglia and neurons, under ischemic conditions. Furthermore, hNSC-EVs loaded with miR-125a-5p (EVs^miR^) promoted the polarization of anti-inflammatory M2 microglia, resulting in altered inflammatory responses and decreased inflammatory cytokine secretion. Additionally, EVs-miR-125a-5p exerts a significant impact on microglia, subsequently translocating to neurons and inhibiting neuronal death. Moreover, increased miR-125a-5p levels in hNSC-EVs effectively inhibited neuronal apoptosis and improved the axonal ultrastructure and neurological function *in vivo*. Mechanistically, EVs^miR^ regulate the TLR4/NF-κB signaling pathway by targeting *IKBKG* to alleviate neuroinflammation induced by CIRI.

**Conclusions:** Our findings demonstrate that miR-125a-5p mechanisms contribute to modulating the neuroinflammatory microenvironment and miR-125a-5p-enriched EVs may be a promising therapeutic strategy for CIRI.

## Introduction

Stroke, a sudden onset of cerebral blood circulation disorder, has become the leading cause of disability and death in China, and is a major cause of sustained and acquired disabilities in adults worldwide 1, 2. The key to treating ischemic stroke is timely restoration of blood perfusion. However, when blood flow to the brain tissue is restored, not only tissue injury but also irreversible damage, such as malignant edema and hemorrhage occurs, leading to a phenomenon known as cerebral ischemia-reperfusion injury (CIRI). Its pathogenesis is complex and typically accompanied by the release of various inflammatory mediators, infiltration and accumulation of inflammatory cells, generation of oxygen free radicals, disruption of the blood-brain barrier (BBB), secretion of inflammatory factors, and massive release of adhesion molecules, which exacerbate brain tissue injury, causing functional disorders and even loss of physiological functions 3. Neuroinflammation is an important pathological feature of CIRI. Therefore, effectively reducing the production of inflammatory factors is crucial for treating such injuries 4.

Microglia, such as macrophages in the central nervous system (CNS), are crucial for regulating immune-inflammatory responses to maintain brain homeostasis 5. The activation of microglia is the initial step in the brain inflammatory response, followed by the infiltration of adaptive immune cells (such as neutrophils, macrophages/monocytes, natural killer cells, and T cells) and activation of other neural cells 6. Moreover, various types of resting neuroglial cells are promptly activated and migrate to the area surrounding the injury, physically enclosing or targeting the lesion areas, which may consist of dead cells, injured neurons, dendrites, blood vessels, or amyloid plaques 7. Microglia, the primary effectors of inflammatory responses, play a crucial role and are typically polarized into the pro-inflammatory M1 and anti-inflammatory M2 phenotypes 8. Modulating the transformation of microglia from M1 to M2 type or promoting the polarization of resting microglia towards the M2 phenotype can alleviate inflammatory responses and improve the prognosis of stroke 9.

Stem cells have certain therapeutic effects on CIRI 10. However, stem cell transplantation has several limitations. First, the survival rate of transplanted stem cells in target tissues is exceedingly low. Second, stem cell transplantation is speculated to cause immune rejection, malignant cell differentiation, and tumorigenesis, which restrict the use of stem cell transplantation for treating CIRI 11. Increasing evidence indicates that stem cells exert potential therapeutic effects via paracrine pathways. Extracellular vesicles (EVs) are among the most important paracrine products that play crucial functional roles 12.

EVs are nanoscale liposomes that originate from the invagination of endosomal membranes and crucial components of cellular paracrine signaling 13. Possessing a lipid bilayer structure, EVs have a diameter of approximately 30-200 nm and carry various intracellular proteins, lipids, mRNAs, microRNAs, and other molecules that play therapeutic roles and participate in complex cell-to-cell signaling 14. Compared with stem cell transplantation, EV transplantation has advantages, such as low immunogenicity, excellent BBB penetration ability, and low vascular occlusion effects, making it suitable for use in CNS injury repair strategies 15. Our preliminary study using an* in vitro* model of ischemia-reperfusion injury revealed that human neural stem cell (hNSC)-derived EVs (hNSC-EVs) inhibit neuronal oxidative injury and apoptosis, and promote neuronal axon elongation and vascular endothelial cell proliferation 16. However, the *in vivo* therapeutic effects of EVs and mechanisms underlying their therapeutic efficacy remain unclear. The activity of EVs is closely related to their components, and miRNAs, as their key components, may be the material basis for its action.

MiRNAs are a highly conserved class of non-coding small RNA molecules consisting of approximately 18-25 nucleotides and mainly produced by the intronic sites of genes. Mature miRNAs can bind complementarily to target molecules, induce target degradation, or inhibit translation, thereby regulating almost all physiological or pathological stages of the body, such as cell growth, development, proliferation, differentiation, and apoptosis 17. Some studies have suggested that miRNAs may be involved in regulating the pathophysiological processes of CIRI and are considered potential diagnostic and prognostic biomarkers for CIRI as well as promising agents for CIRI treatment 18. The expression of miRNAs demonstrates temporal and spatial specificity, with specific miRNAs potentially displaying varying expression levels at different developmental stages or pathological states at the cellular, tissue, and individual levels 19.

**Scheme 1:**
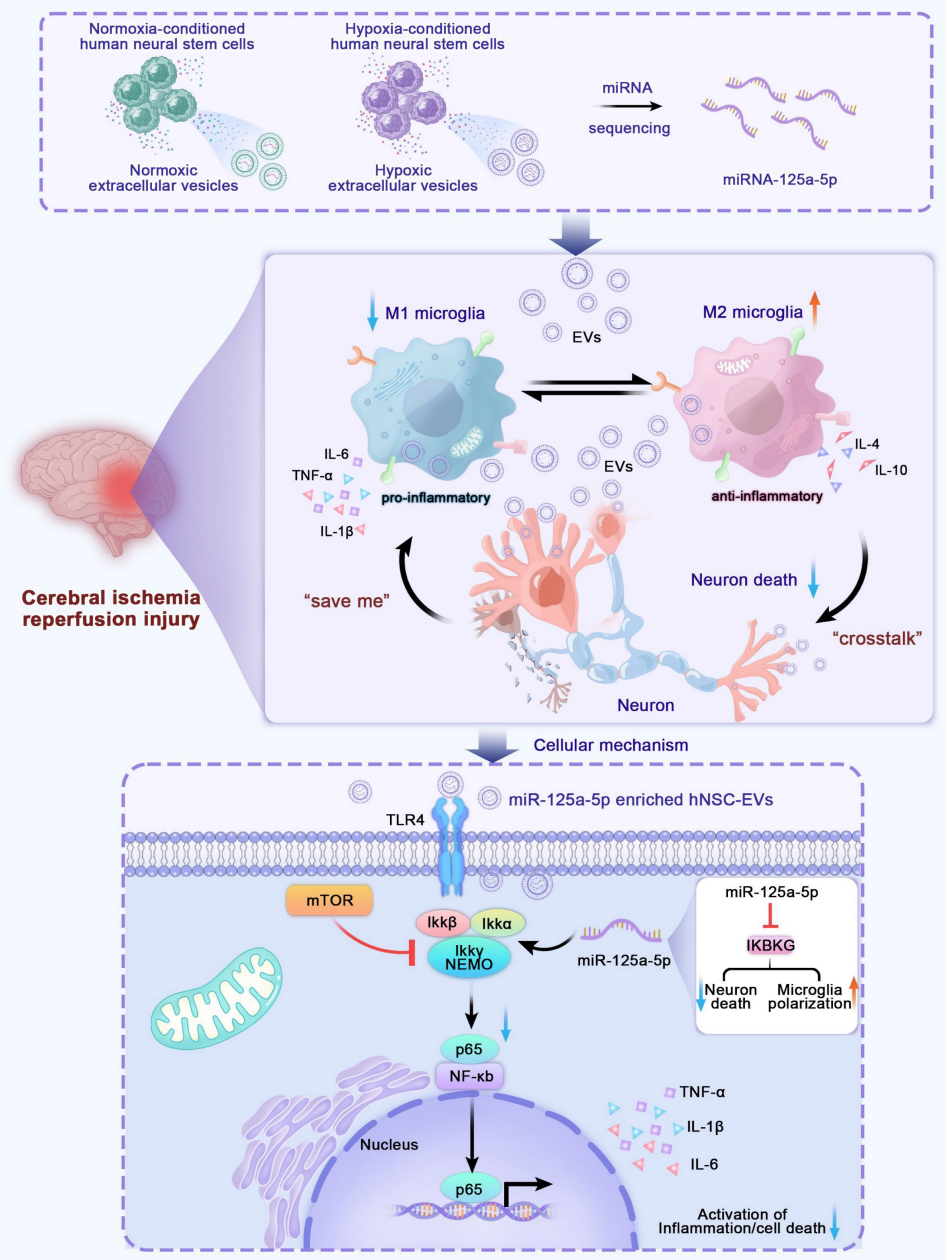
** Scheme of the model of hNSC-EVs accelerates nerve renovation by releasing miR-125a-5p.** After MCAO, the ischemic hNSCs release neural-repair miRNA-enriching EVs, which are transferred to microglia and neurons through the circulatory system and target to mTOR and TLR4/NF-κB pathway to promote neurogenesis and alleviating neuroinflammation. MiR-125a-5p enriched in ischemic hNSC-EVs has potent potential to promote microglia polarization and repair neuron injury by directly targeting *IKBKG*, respectively.

In the present study, we successfully identified miR-125a-5p as a possible crosstalk signal under ischemic conditions by comparing the differences in neural stem cell exosomal miRNAs under normal and hypoxic conditions. We found that EVs carrying miR-125a-5p could effectively alleviate neuroinflammation after CIRI, effectively promote microglia M2 polarization, and inhibit M1 microglial proliferation after late-stage microglial overactivation. Intravenous injection of miR-125a-5p enriched EVs (EVs^miR^) into a middle cerebral artery occlusion (MCAO) rat model promoted the recovery of neurological function by effectively reducing infarcted tissue volume, preventing neuronal death, and improving axonal ultrastructure. More importantly, we demonstrated that miR-125a-5p transmitted by EVs could bind to its target, IκB kinase gamma (IKBKG) protein, thus inhibiting the TLR4/NF-κB pathway to exert anti-inflammatory effects (Scheme 1). Hence, our research offers a proof-of-concept investigation aimed at examining post-stroke inflammatory responses to miR-125a-5p-enriched EVs and the potential of EVs^miR^ for ischemic stroke therapy.

## Methods

### Cell cultures

Human neural stem cells (hNSCs) were donated by Beijing Yinfeng Dingcheng Biotechnology Co., Ltd., (Beijing, CN). The primary hNSCs were isolated specifically from fetal brain tissue with the approval of the Ethics Committee of the Wen'an County Maternity and Child Health Care Hospital. Written informed consent was obtained from each mother prior to tissue procurement. Donated hNSCs were subjected to identification and microbiological safety testing (sterility, mycoplasma, endotoxins, and endogenous and exogenous viral factors). The hNSCs were cultured in serum-free medium DMEM/F12 (Gibco, USA) containing 2 nM L-glutamine (Gibco), 2% B27 supplement (Gibco), 20 ng/mL of human recombinant epidermal growth factor (R&D Systems, USA), 20 ng/mL of basic fibroblast growth factor (R&D Systems), 5 μg/mL of heparin (Sigma-Aldrich, USA) and 1% antibiotic-antimycotic mixture (Gibco), at 37 °C in a humidified atmosphere containing 5% CO_2_. The medium was replaced with fresh medium every 2-3 days. Single cells were separated from neurospheres using Accutase dissociation reagent (Gibco) on the 7th day and resuspended in neuron culture medium at 37 °C under a humidified atmosphere with 5% CO_2_. The hNSCs were maintained in neurobasal medium (Gibco) supplemented with 2% B27 Supplement and 1% L-glutamine for 7 days. The medium was replaced with fresh medium twice a week.

BV2 cells, HT22 cells, and HEK293 cells were purchased from the National Collection of Authenticated Cell Cultures (China). The cells were cultured in DMEM (Biological Industries, Israel) supplemented with 10% FBS, 100 IU/mL of penicillin, and 100 mg/mL of streptomycin sulfate, and then incubated at 37 °C in a humidified atmosphere containing 5% CO_2_.

### Separation and identification of EVs

The hNSC-EVs were isolated from the hNSC culture supernatant using ultracentrifugation as previously described 20. The conditioned medium was collected in a sterile condition and centrifuged at 300 × g for 10 min, 1,000 × g for 15 min, and 10,000 × g for 30 min at 4 °C, and filtered through a 0.22-μm membrane (Millipore, Ireland) to remove non-adherent cells, debris, and large vesicles. The EVs were then isolated using ultracentrifugation at 100,000 × g for 70 min at 4 °C, then washed with phosphate buffer solution (PBS) at 100,000 × g for 70 min at 4 °C. Finally, the pellets were resuspended in cold PBS and stored at -80 °C.

Nanoparticle tracking analysis (NTA) was performed to measure the effective diameter and size distribution of EVs using a ZetaVIEW instrument (Particle Metrix, Germany). The equipment was calibrated using 100-nm polystyrene beads with a 405-nm excitation laser, and EVs were diluted in sterile-filtered PBS (1:1000).

The structure of hNSC-EVs was observed using transmission electron microscopy (TEM) (Talos, USA). EV solution (5 μL) was loaded onto a copper grid with a 500 mesh carbon film (Electron Microscopy Sciences, Washington, PA, USA). The sample was contrast-stained on the grid with a drop of 2% uranyl acetate solution. Excess liquid was removed using absorbent paper and dried with air. EV images were recorded using TEM at an acceleration voltage of 80 kV and exposure time of 100 ms.

The surface markers of the EVs were measured with ExoView (NanoView Biosciences, MA, USA) 21. The sample concentration was diluted to 10^5^-10^8^ particles/mL using an ExoView tetraspanin kit. The diluted EVs were carefully pipetted onto silicon chips coated with individual antibody spots against mouse CD9, CD63, and CD81 (1:1000, 251-1044, Leprechuan) as well as negative isotype controls. After overnight incubation, the chips were washed thrice on a shaker with PBS containing 0.1% Tween-20. The chips were then incubated with fluorescent antibodies (anti-human CD81 conjugated to Alexa Fluor 488, CD63 conjugated to Alexa Fluor 647, and CD9 conjugated to Alexa Fluor 555) for 1 h at room temperature. Image and data acquisition for each chip was performed with the ExoView R100 (NanoView Biosciences).

### Preparation of oxygen-glucose deprivation (OGD) model

To simulate ischemia-like conditions, hNSCs were cultured for 3 days and the culture medium was replaced with glucose-free DMEM (Gibco). Then the hNSCs were incubated in an oxygen-deprived (1% O_2_, 5% CO_2_, and 94% N_2_) incubator for 2 h at 37 °C. Next, glucose-free DMEM was replaced with normal medium and the cells were returned to an incubator under normal conditions for 24 h.

Neurons and BV2 cells were exposed to the OGD environment *in vitro* to mimic the ischemia-like conditions. Glucose-free DMEM was added to the cells to replace the culture medium and the cells were incubated in an anoxic incubator with 1% O_2_, 5% CO_2_ and 94% N_2_ at 37 °C for 2 h. The cells were then placed under normal conditions and subjected to reperfusion for 24 h.

### Axonal elongation assay

Neuronal cells were fixed with 4% paraformaldehyde, permeabilized with 0.1% Triton X-100 (Sigma-Aldrich), and blocked with 5% bovine serum albumin (BSA; Solarbio). The cells were incubated overnight at 4 °C with microtubule-associated protein 2 (MAP-2; 1:5000, ab5392, Abcam) primary antibody and then incubated with Alexa Fluor 488-conjugated secondary antibody (Abcam) for 1 h at room temperature. The nuclei were labeled with Hoechst 33342 (Proteintech) and examined using an Opera Phenix screening system (PerkinElmer, USA). The lengths of the axons were analyzed using ImageJ (version 1.52a).

### Angiogenesis assay

Matrigel (50 μL; BD Bioscience, USA) was placed in each well of a 96-well plate and incubated for 1 h at 37 °C. Under OGD conditions, 2 × 10^4^ human umbilical vein endothelial cells (HUVECs) were inoculated onto Matrigel and cultured in a glucose-free medium for 2 h in an anoxic incubator. The medium was replaced with normal medium containing EVs or hypoxic EVs. The HUVECs were then incubated at 37 °C with 5% CO_2_ for 6 h. Angiogenesis in control and EVs or hypoxic EVs groups was photographed using a microscope (Nikon, Japan) and analyzed using ImageJ.

### Sequencing of EV miRNAs

Total RNA from EVs was extracted using the miRNeasy Kit (Qiagen, Hilden, Germany) according to the manufacturer's instructions. The extracellular fractions of EVs generated from hNSCs and OGD-preconditioned hNSCs were sequenced in triplicate. According to the Qiagen QIAseq miRNA Library Kit strategy, the miRNA sequencing library was constructed with 18-30 nt miRNAs of total RNA by performing a 3′-end linker, a 5′-end linker, reverse transcription, and PCR. The cDNA libraries were submitted to RNA sequencing on an Illumina HiSeq sequencer platform.

### Dual luciferase reporter gene assay

The TargetScan 7.2 (targetscan.org) was used to predict the target genes of miR-125a-5p, and a dual-luciferase reporter assay was used to verify whether *IKBKG* was the direct target gene of miR-125a-5p. To construct luciferase reporter vectors, the 3′-untranslated region (3′-UTR) of wild-type *IKBKG* containing a predicted miR-125a-5p binding site and mutant *IKBKG* 3′-UTR with no miR-125a-5p binding site were used to generate Luc-*IKBKG*-wt and Luc-*IKBKG*-mut vectors using PmirGLO (Promega, USA) as the backbone. For reporter assays, HEK293T cells were cultured at 2.5 × 10^4^ cells/well in 24-well plates and cotransfected with the miR-125a-5p mimics or miRNA mimics negative control (50 nM), and 0.2 µg of the Luc-*IKBKG*-wt or Luc-*IKBKG*-mut vector. The transfection was performed using Lipofectamine 3000 (Invitrogen, USA). After 48 h post-transfection, luciferase activity was measured using a Centro LB963 (Berthold, USA) and the Dual-Luciferase Reporter Assay System kit (Promega). Firefly luminescence was normalized to Renilla luminescence to calculate the relative luciferase activity.

### Loading of small RNA into hNSC-EVs using electroporation

Approximately 10^10^ total EV particles and 10 μg of miRNA mimics (EVs^miR^) or negative controls (EVs^NC^) were lightly mixed in 400 μL of cold electroporation buffer (1.15 mM potassium phosphate, 25 mM potassium chloride, and 21% Optiprep, pH 7.2). The mixture was electroporated in ice-cold 4-mm cuvettes under the following conditions: 400 V, 125 μF, and ∞ Ω using a Gene Pulser Xcell Electroporation System (BioRad, USA) as previously described 22. To recover the complete membrane structure, the mixture was incubated at 37 °C for 30 min. After incubation, the EV samples were washed with cold PBS and centrifuged at 100,000 × g for 70 min at 4 °C to remove unloaded miR-125a-5p.

### MiRNA extraction and reverse transcription reaction

MiRNAs were extracted from the EV samples using the Qiagen miRNeasy Mini Kit (Qiagen). A 200 nM spike-in control (miR-39) was added during isolation of the RNA fraction as an extraction control. The concentration and purity of RNA were determined using a NanoDrop-2000 spectrophotometer (Thermo Fisher Scientific, USA). Reverse transcription and quantitative PCR of miRNA samples were performed on a lightCycler480 Real-Time PCR Detection instrument (Roche, Switzerland) using the miDETECT A Track miRNA qRT-PCR Starter Kit (RiboBio, China) together with specific miRNA primers (RiboBio). The relative expression levels of miRNAs were calculated using the comparative Ct (2^-ΔΔCt^) method.

### Droplet digital PCR (ddPCR)

Targeted miRNAs were reverse transcribed using 100 ng of total RNA, sequence-specific miDETECT A Track miRNA RT primers, and the miDETECT A Track miRNA qRT-PCR Starter Kit (RiboBio). RNA levels were quantified in absolute terms using a QIAcuity Digital PCR System (Qiagen). The thermal cycling program consisted of 2 min at 95 °C, 40 cycles of 15 s at 95 °C, 15 s at 60 °C, 15 s at 72 °C, and then 5 min at 40 °C. Different fluorescent signals from each partition were detected and the absolute copy number of miRNAs was determined using Poisson distribution with the QIAcuity software.

### MiRNA transfection

BV2 cells were cultured to 70-80% confluence and transfected with miR-125a-5p mimic (sense, 5′-UCCCUGAGACCCUUUAACCUGUGA-3′), miR-181a-5p mimic (sense, 5′-AACAUUCAACGCUGUCGGUGAGU-3′), miR-27a-3p mimic (sense, 5′-UUCACAGUGGCU AAGUUCCGC-3′), miR-21-5p mimic (sense, 5′-UAGCUUAUCAGACUGAUGUUGA-3′), miR-92b-5p mimic (sense, 5′-UAUUGCACUCGUCCCGGCCUCC-3′), miR-4644 mimic (sense, 5′-UGGAGAGAGAAAAGAGACAGAAG-3′), miR-125a-5p inhibitor mimic (sense, 5′-UCACAGGUUAAAGGGUCUCAGGGA-3′) and miRNA mimic negative control (sense, 5′-UUCUCCGAACGUGUCACGUTT-3′) (GenePharma Inc, Shanghai, China) with Lipofectamine RNAiMAX (Invitrogen), according to the manufacturer′s instructions. MiRNA mimics and miRNA mimic negative controls were used at a final concentration of 50 nM and incubated for 6 h. Subsequently, the medium was replaced with normal culture medium to terminate transfection.

### qPCR

Total cellular RNA was isolated from cells using the RNeasy Plus Mini kit (Qiagen) according to the manufacturer's guidelines. RNA concentration and quality were evaluated using a NanoDrop-2000 spectrophotometer. Equal quantities (1 μg) of isolated RNA were converted to cDNA using reverse transcription with a Fastking RT kit (TIANGEN, China) and quantitative real-time PCR was performed using a TB Green Premix Ex Taq kit (TaKaRa, Japan) on a lightCycler480 Real Time PCR system (Roche, Switzerland). GAPDH was used as the internal control and all values were calculated using the 2^-ΔΔCt^ method. The primer sequences for the detection of TNF-α, IL-1β, IL-6, IL-10, TGF-β, Arg-1, iNOS, CD16, and CD206 were as follows:

TNF-a: CCCTCACACTCAGATCATCTTCT (forward), GCTACGACGTGGGCTACAG (reverse); IL-1β: GCAACTGTTCCTGAACTCAACT (forward), ATCTTTTGGGGTCCGTCAACT (reverse); IL-6: TAGTCCTTCCTACCCCAATTTCC (forward), TTGGTCCTTAGCCACTCCTTC (reverse); IL-10: GCTCTTACTGACTGGCATGAG (forward), CGCAGCTCTAGGAGCATGTG (reverse); TGF-β: TCTGCATTGCACTTATGCTGA (forward), AAAGGGCGATCTAGTGATGGA (reverse); Arg-1: CTCCAAGCCAAAGTCCTTAGAG (forward), AGGAGCTGTCATTAGGGACATC (reverse); iNOS: GTTCTCAGCCCAACAATACAAGA (forward), GTGGACGGGTCGATGTCAC (reverse); CD16: CAGAATGCACACTCTGGAAGC (forward), GGGTCCCTTCGCACATCAG (reverse); CD206: CTCTGTTCAGCTATTGGACGC (forward), CGGAATTTCTGGGATTCAGCTTC (reverse); and GAPDH: AGGTCGGTGTGAACGGATTTG (forward), TGTAGACCATGTAGTTGAGGTCA (reverse).

### Coculture experiments

The well inserts with a 0.4-mm pore size filter (BD Falcon, Corning, NY, USA) for 24-well plates were used following the manufacturer′s instructions. BV2 cells were seeded into well inserts containing DMEM. HT22 cells were seeded in 24-well plates. After OGD treatment, BV2 cells were washed with PBS and co-cultured with HT22 cells for 24 h according to the experimental protocol.

### Cell counting kit-8 assay

Cell viability was determined using the Cell Counting Kit-8 assay (CCK-8, APExBIO, USA). BV2 or HT22 cells were exposed to OGD conditions for 2 h and then treated with miRNA mimics, EVs^NC^, and EVs^miR^. The supernatant was discarded and medium containing CCK-8 solution was incubated for 1 h at 37 °C. The absorbance was measured at 450 nm using a microplate reader. The results were assayed in triplicate.

### Preparation of the ischemia animal model MCAO

Male Sprague-Dawley (SD) rats weighing 280-320 g (10-12 weeks) purchased from Vital River Laboratory (Beijing, China) were used in all studies. All rats were individually kept under specific pathogen-free conditions, and maintained at an ambient temperature of 20-26 °C and a humidity of 40-70% on a 12 h light-dark cycle in the Model Animal Research Center of Shandong University. The experiment was conducted after a week of adaptive feeding. All animal studies were performed in accordance with the National Institutes of Health Animal Care and Use Guidelines and approved by the Ethical Committee of the School of Pharmaceutical Sciences, Shandong University (approval number 22017).

The rats were fasted for 12 h and deprived of water for 4 h before surgery. CIRI was induced 2 h after middle cerebral artery occlusion. In the main study, the rats were anesthetized with 3% isoflurane and maintained with 2.0% isoflurane. The right common carotid artery, internal carotid artery (ICA), and external carotid artery were separated using a ventral neck incision. A 0.38-mm commercial silicon-coated monofilament with a rounded tip was gently inserted into the right ICA to block the right middle cerebral artery (MCA). After 2 h of MCAO, reperfusion was performed by withdrawing the monofilaments.

### Treatments and experimental protocols

The rats were randomly divided into four groups: sham, MCAO, EVs^NC^, and EVs^miR^. After reperfusion, the rats were slowly administered PBS (for the sham and MCAO groups), EVs^NC^, or EVs^miR^ diluted in 1.5 mL (1×10^11^) of PBS via tail vein injection. To assess the recovery of neurological function, a gait test and Zea-Longa scoring were performed after treatment. Behavioral tests were performed on days 3 and 7. Infarct volume and microstructural changes in the brain were measured using magnetic resonance imaging (MRI).

### Neurological function assessments of neurological deficits

On the 3rd and 7th days after MCAO injury, the researcher, who was blinded to the grouping, conducted neurological function assessments. Neurological function assessments included Zea-Longa scoring, rat gait analysis, and grasping capability tests to evaluate motor function recovery in rats. Neurological function assessments were performed on 8-10 rats in each group.

### Zea-Longa scoring

Zea-Longa scoring consisted of 5 grades: 0 points, no neurological deficit; 1 point, mild neurological deficit, left front paw not fully extended; 2 points, moderate neurological deficit, turning to the left circle; 3 points, severe neurological deficit, dumping to the left (paralyzed side); and 4 points, inability to walk spontaneously, loss of consciousness.

### Rat gait analysis

To assess the recovery of motor function, rats were objectively evaluated using the CatWalk XT system (Noldus, Netherlands) after MCAO reperfusion (MCAO/R) injury. One week before the test, the rats were trained to cross the glass platform at least three times. Gait analysis was performed in a dark and quiet environment on the 3rd and 7th days after MCAO/R. Under free walking conditions, the pawprints of the rats were captured using a high-speed camera at the bottom of the platform. The right forelimb, right hindlimb, left forelimb, and left hindlimb were automatically labeled using gait parameters. The stance ratio, swing ratio, brake ratio, paw area, and stride length of the gait parameters were analyzed using CatWalk XT software (version 10.6, Noldus).

### Grasping capability test

A 40-cm rope was placed 70 cm from the ground and a foam box was positioned beneath it. One week before the test, the rats were trained to grasp the rope at least three times. The forelimbs of the rats were suspended from the rope and released. The time required by the rats to grasp the rope before landing was recorded.

### MRI acquisition and analysis

MRI was routinely used to detect cerebral ischemia in clinical settings. MRI measurements were performed using a 9.4 T small animal MRI scanner (Bruker Biospin, Germany) equipped with Paravision 6.0.1 software (Bruker Biospin). The rats were anesthetized with 5% isoflurane and maintained spontaneously with 2.0% isoflurane. The respiratory rates of the rats were constantly monitored during MRI scanning. On the 3rd and 7th days post surgery, the rats underwent MRI sessions, including T2-weighted imaging (T2WI), diffusion-weighted imaging (DWI), T2 mapping, two-dimensional time-of-flight (TOF) magnetic resonance angiography (2D-TOF MRA), and diffusion tensor imaging (DTI).

T2WI was used to calculate the lesion volume in MCAO rats. The parameters of T2WI were acquired using a spin-echo sequence as follows: Echo Time/Repetition Time (TE/TR) = 33/2500 ms, field of view (FOV) = 34 × 32 mm, matrix size = 256 × 256, and slice thickness = 0.8 mm. Regions of hyperintensity on the T2 scans were used to determine the infarct regions. Twenty-five consecutive coronal slices were acquired, covering the entire extent of the rat brain without any gaps between slices. For each rat brain, the area of the stroke lesion and total brain area in every slice were manually measured using the ImageJ software. Total stroke volume was calculated as the sum of the lesion area across all slices, multiplied by the slice thickness (0.8 mm). Similarly, the total brain volume was calculated by summing the areas of each brain slice and multiplying them by the slice thickness. The infarct volume ratio was calculated as the total infarct volume divided by the total brain volume.

DWI was conducted to detect the random Brownian motion of water molecules within the tissues. The DWI sequence was acquired using the following parameters: TE/TR = 18/2500 ms, FOV = 33 × 30 mm, b-value = 0, 1400 s/mm^2^, matrix = 128 × 128, and slice thickness = 0.8 mm. Apparent diffusion coefficient (ADC) maps were generated using Paravision version 6.0.1 software. ADC values were determined from identical consecutive DWI images.

T2 mapping was used to determine the structural changes in the tissue lesions. The sequence was acquired use the following parameters: TR = 5262 ms, TEs from 7.5 to 90 ms, FOV = 35 × 28 mm, matrix size = 192 ×192, and slice thickness = 0.8 mm. The peri-infarct cortex and striatum were drawn as regions of interests (ROIs). The T2 values were acquired using coronal T2 relaxometry maps of the ROIs. The ratio of ipsilateral T2 values to contralateral T2 values is presented as a relative value.

The 2D-TOF MRA was performed to detect changes in the intracranial arteries. The sequence was acquired using the following parameters: TE/TR = 1.9/12 ms, FOV = 28 × 30 mm, matrix size = 256 × 256, and slice thickness = 0.4 mm. The images of 2D-TOF MRA were reconstructed using Paravision version 6.0.1 software. The 2D-TOF MRA was conducted to evaluate the MCA, ICA, bilateral anterior cerebral artery, anterior communicating cerebral artery, anterior azygos cerebral artery, posterior cerebral artery, and basilar artery in the peri-infarct tissue.

DTI was used to detect nerve fiber injury in the peri-infarct tissue using a multislice spin-echo sequence. The sequence parameters were as follows: TE/TR = 22/2000 ms, FOV = 21 × 31 mm, matrix size = 128 × 128, slice thickness = 0.8 mm, 30 diffusion directions, and b values = 0, 600, 1000, and 1400 s/mm^2^. ROIs of DTI were placed in the same areas as the T2 relaxometry mapping images. Fractional anisotropy (FA), axial diffusivity (AD), radial diffusivity (RD), and mean diffusivity (MD) maps were constructed using Paravision version 6.0.1 software. The FA, AD, RD, and MD values were determined using DTI parametric maps.

### 3D MRI processing

Image data were resampled to facilitate more accurate brain extraction using a ResampleImage tool in Advanced Normalization Tools. Subsequently, the 3dSkullstrip tool of AFNI (Analysis of Functional NeuroImages) was used for rat brain extraction. To separate stroke regions from the normalized brain data, a 3D slicer segmentation wizard toolkit was used. Segmented stroke regions were overlaid on the rat brain to be visualized in the 3D space using the Multi-Image Analysis GUI (Mango).

### Triphenyltetrazolium chloride (TTC) staining

TTC staining was conducted to measure the infarct size of the brain tissue and examine tissue viability after MCAO/R injury. Rats were deeply anesthetized and perfused with ice-cold saline containing 10 U/mL of heparin. The whole brain tissue was carefully removed and kept at -20 °C for 20 min. The brains were carefully dissected into 2 mm-thick coronal sections, then incubated in a 2% 2,3,5-triphenyltetrazolium chloride (TTC, Sigma-Aldrich) solution for 20 min at 37 °C. Subsequently, the brain slices were immersed in 4% paraformaldehyde overnight and photographed using a camera. Infarct volumes were analyzed using ImageJ software.

### Terminal-deoxynucleoitidyl transferase dUTP nick end labeling (TUNEL) staining

TUNEL staining was used to evaluate apoptotic cells in the MCAO injury region using the TUNEL reagent (Roche, Switzerland). The paraffin sections were baked for 10 min, dewaxed with xylene, treated with 3% H_2_O_2_, and incubated in 0.1 M sodium citrate. The sections were stained with diaminobenzidine after incubation with the TUNEL reaction mixture. DAPI (4ʹ,6-diamidino-2-phenylindole) was used to label the nuclei of neurons.

### Western blotting

Total protein was extracted using by RIPA lysis buffer (Beyotime, China). The total protein concentration was measured using a Bicinchoninic Acid protein assay kit (Thermo Fisher Scientific). Total protein (30 μg) was loaded and subjected to 10% SDS-PAGE, then transferred to polyvinylidene difluoride (PVDF) membranes (Millipore, USA). After blocking with 5% BSA for 2 h, the membranes were incubated with corresponding primary antibodies, including p-NF-κB (1:1000, 3033, Cell Signaling Technology), NF-κB (1:1000, 8242, Cell Signaling Technology), p-mTOR (1:1000, 5536, Cell Signaling Technology), mTOR (1:1000, 2983, Cell Signaling Technology), TLR-4 (1:500, sc-293072, Santa Cruz), IKBKG (1:1000, 18474, Proteintech), and GAPDH (1:5000, ab8245, Abcam) at 4 °C overnight. The membranes were washed three times with Tris-buffered saline containing Tween 20, followed by incubation with HRP-conjugated Goat Anti-Rabbit IgG(H+L) (1:5000, SA00001-2, Proteintech) or HRP-conjugated Goat Anti-Mouse IgG(H+L) secondary antibody (1:5000, SA00001-1, Proteintech). The PVDF membranes were developed by adding enhanced chemiluminescence reagents, visualized using an Amersham Imager 600 (GE Healthcare Life Sciences, USA), and analyzed using ImageJ software.

### Enzyme-linked immunosorbent assay (ELISA)

On the 3rd day after MCAO, brain tissues were collected from the injury and EV treatment groups to evaluate the inflammatory response in injured neurons. The lysates of the brain tissues were isolated and centrifuged at 5,000 × g for 5 min at 4 °C. The supernatants were used to determine the expression levels of anti-inflammatory cytokines, including IL-4 and IL-10, and pro-inflammatory cytokines, including TNF-α, IL-1β, and IL-6, using ELISA kits (Elabscience, IL-4, R0014c; IL-10, R0016c; TNF-α, R2856c; IL-1β, R0012c; IL-6, R0015c). Absorbance was measured at 450 nm using a multimode plate reader (PerkinElmer).

In BV2 culture medium, the pro- and anti-inflammatory cytokines were measured using ELISA kits, according to the manufacturers' protocols (Elabscience). The concentrations of inflammatory cytokines are presented as pg/mL.

### Immunofluorescence staining and immunohistochemistry analysis

Brain tissue was collected, fixed in 4% paraformaldehyde, and embedded in paraffin. Paraffin sections of brain tissue were baked, deparaffinized with xylene, and immersed in graded ethanol. The sections were washed three times with PBS and blocked with 5% BSA at 37 ºC for 2 h. Histological changes in brain cells were examined using hematoxylin-eosin staining. Neuronal structures were observed using Nissl staining. Finally, the brain sections were photographed under a panoramic scanning and image analysis system (Olympus, Tokyo, Japan).

For immunofluorescence staining, brain slices were permeabilized with 0.3% or 0.1% Triton X-100 and blocked with 5% BSA (Sigma-Aldrich). Brain sections were then incubated with primary antibodies. To evaluate the polarization of microglia, the primary antibody against iNOS (1:300, sc7271, Santa Cruz) was used to label M1 microglia and the primary antibody against mannose (CD206) (1:1000, ab64693, Abcam) was used to label M2 microglia. To detect anti-inflammatory effects, paraffin sections of brain tissues were stained and labeled with glial fibrillary acidic protein (GFAP) antibody (1:1000, ab7260, Abcam) for astrocytes, Iba1 antibody (1:100, Abcam) for microglia, and NeuN antibody (1:1000, ab104224, Abcam) for neurons. The primary antibody of NF-κB (1:600, 8242, CST) was used to access the location of NF-κB factor expression. After washing twice with PBS, secondary antibodies were added to the slices, including Alexa Fluor 488-conjugated Donkey anti-goat IgG(H+L) (1:600, ab150133, Abcam), Alexa Fluor 555-conjugated Donkey anti-mouse IgG(H+L) (1:600, ab150110, Abcam), and Alexa Fluor 647-conjugated Donkey anti-rabbit IgG(H+L) (1:600, ab150063, Abcam), followed by DAPI staining (2 μM, Sigma-Aldrich). Images were captured using a confocal laser-scanning microscope (Olympus). Images were processed using the ImageJ software.

### Statistical analysis

All statistical analyses were performed using the GraphPad Prism 6.0 software (GraphPad Software, San Diego, CA, USA). Immunostaining images were analyzed using ImageJ Software. *P*-value was determined using Student's *t*-test for two-group comparisons or one-way ANOVA for multiple group comparisons. *P* < 0.05 was considered to be statistically significant. Data acquired from multiple experiments were presented as the mean ± SEM.

## Results

### MiR-125a-5p is a key mediator of the neuroinflammatory modulation and neural restoration of hNSC-EVs

**Figure 1:**
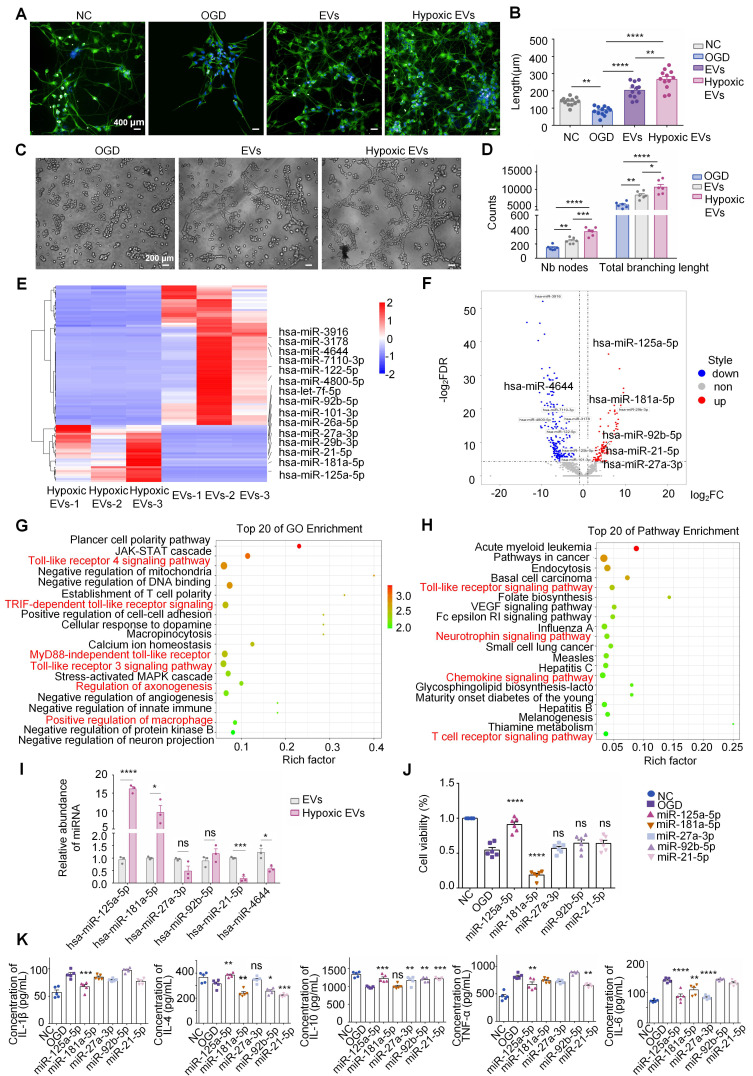
** Identification of differential miRNAs between normal-conditioned human neural stem cell-extracellular vesicles (hNSC-EVs) and oxygen-glucose deprivation (OGD)-preconditioned hNSC-EVs.** (A) Observation of neurites in neurons in normal control (NC), OGD, EVs, and hypoxic EVs groups using the Opera Phenix screening system. Scale bars, 400 μm. MAP-2 (green) and Hoechst 33342 (blue). (B) Quantitative analysis of the neurite length in NC, OGD, EVs, and hypoxic EVs groups using ImageJ. Total lengths of neurites per neuron are measured (*n* = 12). (C) Observation of angiogenesis of human umbilical vein endothelial cells (HUVECs) in OGD, EVs, and hypoxic EVs groups using a microscope under OGD condition (×100). Scale bars, 200 μm. (D) Quantitative analysis of the number of nodes (NB) and total branching length of angiogenesis in OGD, EVs, and hypoxic EVs groups using ImageJ (*n* = 6). (E) Heatmap of representative miRNAs with a ≥ 2-fold difference and *P* values ≤ 0.05 between the normal and OGD-preconditioned hNSC-EVs. (F) Scatter plot of miRNA differential expression analysis between samples. Red dots indicate significantly upregulated miRNAs and blue dots indicate significantly downregulated miRNAs. (G-H) Gene Ontology (GO) and Kyoto Encyclopedia of Genes and Genomes pathway (KEGG) enrichment analysis between samples. The size of the node represents the number of genes and the color of the node represents the corresponding *P-*value. (I) Comparison of the relative contents of miR-21-5p, miR-27a-3p, miR-92b-5p, miR-125a-5p, miR-181a-5p, and miR-4644 between normal- and OGD-preconditioned hNSC-EVs at 24 h post-impact using qRT-PCR (*n* = 3). (J-K) The BV2 cells were transfected with miRNA or negative control mimics. The CCK-8 assay and ELISA were performed to analyze inflammatory response in BV2 cells after transfection. (*n* = 6 in CCK-8;* n* = 5 in ELISA). The quantitation results were plotted as dot plots, showing the mean ± SEM of independent experiments. *P < 0.05, **P < 0.01, ***P < 0.001, ****P < 0.0001; ns: not significant.

Recently, it was demonstrated that preconditioning with hypoxia may alter the miRNA profile of EVs and improve the neuroprotective effects of miRNA-enriched EVs administered to recipients 23. Figure 1A-D show that EVs derived from hypoxia-preconditioned hNSCs had a much greater potential to promote axon elongation, neuron proliferation, and angiogenesis *in vitro* than those derived under normoxic conditions, which might also be a result of the differences in exosomal miRNAs in EVs. To identify the crucial differentiated miRNAs in EVs and explore their mechanisms, we isolated RNA from normal hNSC-EVs and hypoxia-preconditioned hNSC-EVs, analyzed the miRNAs extracted from the EVs using high-throughput sequencing, and compared them between the two groups. The miRNA analysis showed that 433 miRNAs were differentially expressed (148 upregulated and 285 downregulated) in the hypoxic EVs group compared with their expression in the normal EVs group (log2|fold change|≥2, *P* < 0.05), indicating that miRNAs had distinct expression patterns between the two groups (Figure 1E-F).

In addition, we performed Gene Ontology (GO) and Kyoto Encyclopedia of Genes and Genomes (KEGG) enrichment analyses to identify potentially involved biological processes and pathways. Target gene prediction and enrichment analysis of the differentially expressed miRNAs between normoxic and hypoxic hNSC-EVs were performed. Consequently, the biological process results of GO analysis (Figure 1G) showed that these differentiated miRNAs mainly regulated the JAK-STAT cascade, Toll-like receptor (TLR) 4 signaling pathway, and Toll-receptor-associated activator of interferon (TRIF)-dependent TLR signaling pathway, which were associated with the development of inflammatory responses 24, 25. Some biological processes directly related to the immune response and inflammation 26-28 were also involved, such as the establishment of T cell polarity, positive regulation of cell-cell adhesion mediated by cadherin, regulation of the innate immune response, positive regulation of macrophage activation, and regulation of protein kinase B signaling. Some are directly involved in the regulation of axonogenesis, macropinocytosis, calcium ion homeostasis, and angiogenesis 29. The majority of the concerned biological processes were known to have regenerative or positive effects. In addition, gene enrichment analysis of the EVs based on KEGG was shown in Figure 1H, providing the top 20 enriched pathways of EVs and hypoxic EVs for comparison. KEGG analysis revealed that these genes were enriched in inflammatory responses and neurorestoration-related pathways, such as the TLR, VEGF, chemokine, and neurotrophin signaling pathways. These results suggest that these genes are primarily involved in neuroinflammation during CIRI.

Based on the miRNA profiling data (Figure 1E-F), five differentially upregulated miRNAs (miR-27a-3p, miR-21-5p, miR-92b-5p, miR-125a-5p, and miR-181a-5p) and one downregulated miRNA (miR-4644) were selected, and their expression was validated in the two groups of EVs using qRT-PCR. The hypoxic EVs from human neural stem cells contained higher levels of miR-125a-5p and miR-181a-5p, and lower levels of miR-21-5p and miR-4644 than those of normoxic EVs, whereas the other two pre-selected circulating miRNAs showed no significant differences (Figure 1I). To determine the effects of these miRNAs on neuroinflammation, BV2 cells were transfected with the miRNAs or negative control mimics. Notably, miR-125a-5p significantly promoted the proliferation of BV2 cells, whereas miR-181a-5p had the opposite effect (Figure 1J). We further examined the release of inflammatory factors, and found that miR-125a-5p most significantly reduced the expression of pro-inflammatory factors, TNF-α, IL-1β, and IL-6, and promoted the expression of anti-inflammatory factors, IL-10 and IL-4, among the selected miRNAs (Figure 1K). This suggests that miR-125a-5p significantly reduced neuroinflammatory responses and promoted the proliferation of microglia after hypoxic injury. Moreover, previous studies have indicated that miR-125a-5p is associated with acute ischemic stroke and could potentially serve as a valuable diagnostic biomarker in the early stages of clinical assessment 30. Based on these results, miR-125a-5p was selected as a candidate miRNA for further research.

To further explore the target genes of miR-125a-5p that regulate the immuno-inflammatory response in CIRI, a gene interaction network of miR-125a-5p was built (Figure S1). MiRNA-gene interaction network analysis suggested that miR-125a-5p is a key modulator of gene transcription in hNSC-EVs. From the network, we found that *IKBKG* was the only qualified gene that correlated with TLR4 signaling and targeted by miR-125a-5p. IKBKG recruits the active catalytic inhibitor of the IκB kinase (IKK) component, phospho-IKKβ, to initiate NF-κB signaling and upregulate inflammatory cytokines 31. More importantly, the NF-κB signaling pathway plays a key role in regulating the neuroinflammatory response downstream of the TLR4 signaling pathway 32. The potential role of miR-125a-5p in modulating the TLR4 signaling pathway was investigated in a subsequent neuronal inflammation study to clarify the function and mechanism of EVs after CIRI.

### Preparation and characterization of miR-125a-5p-enriched hNSC-EVs

**Figure 2:**
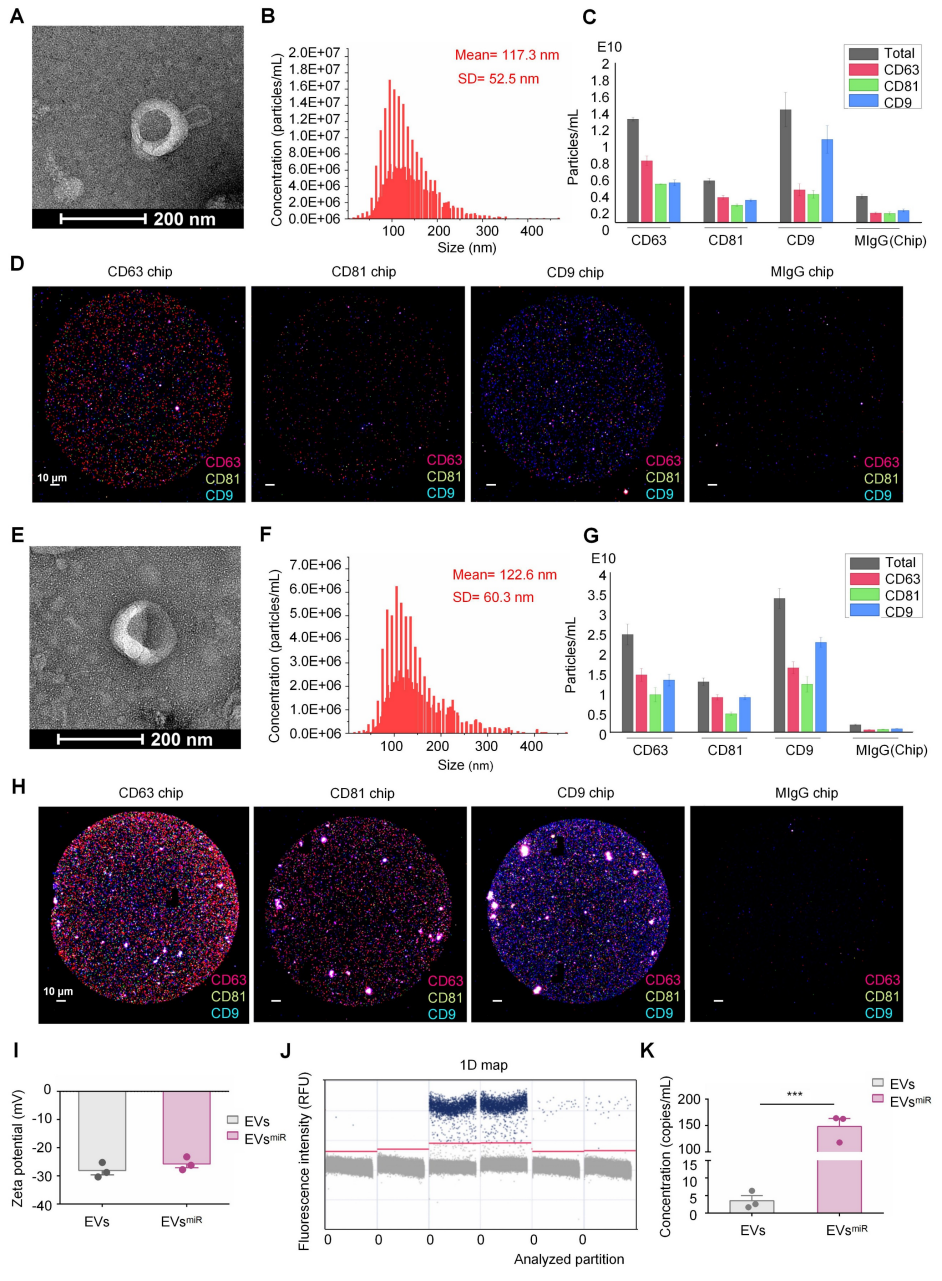
** Preparation and characterization of human neural stem cell extracellular vesicles (hNSC-EVs)^miRNA^.** Transmission electron microscopy (TEM) images of hNSC-EVs (A) and hNSC-EVs^miRNA^ (E), scale bar = 200 nm; particle sizes of hNSC-EVs (B) and hNSC-EVs^miRNA^ (F); representative particle counts on different capture spots from a single chip of hNSC-EVs (C) and hNSC-EVs^miRNA^ (G); representative colocalization images of CD9, CD63 and CD81 on single hNSC-EVs (D) and hNSC-EVs^miRNA^ (H) at different capture spots, mouse IgG (MIgG) were used as the negative markers.; zeta potential (I) of hNSC-EVs and hNSC-EVs^miRNA^ (*n* = 3 independent samples). (J) The separation between positives and negatives in the PCR system. A minimum of 1.0 μL of the assay is recommended for the continuous run. (K) Analysis of the concentration of miR-125a-5p in EVs and EVs^miRNA^ (*n* = 3 independent samples). The quantitation results were plotted as dot plots, showing the mean ± SEM of independent experiments. ***P < 0.001.

HNSC-EVs were isolated separately from the culture supernatants of normal-conditioned hNSCs using the ultracentrifugation method. The morphology and characteristics of the purified hNSC-EVs were examined using TEM, particle size analysis, protein marker detection, and zeta potential analysis. The hNSC-EVs exhibited a typical cup-shape morphology (Figure 2A). NTA revealed that the mean diameter of the hNSC-EVs was approximately 117.3 ± 52.5 nm (Figure 2B). Furthermore, ExoView analysis showed that hNSC-EVs expressed characteristic proteins of EVs, such as CD9, CD63, and CD81 (Figure 2C-D). The surface zeta potential of the hNSC-EVs was slightly fluctuated around -28.74 ± 0.96 mV (Figure 2I).

The miR-125a-5p riched hNSC-EVs (hNSC-EVs^miR^, expressed as EVs^miR^) were prepared by loading miR-125a-5p mimics into hNSC-EVs using the electroporation technology 33. The morphology of EVs^miR^ was similar to that of hNSC-EVs with a saucer-like morphology (Figure 2E). The average diameter of the EVs^miR^ was 122.6 ± 60.3 nm (Figure 2F). ExoView results confirmed that the specific protein markers CD9, CD81, and CD63 were also expressed in EVs^miR^ (Figure 2G-H). The zeta potential of the EVs^miR^ recorded using NTA was -23.20 ± 0.90 mV (Figure 2I). The morphology of EVs^miR^ showed several minor variations compared with that of hNSC-EVs, including a slightly larger average diameter and higher surface potential. ddPCR was chosen to determine the miRNA quantitation of loaded miR-125a-5p. As the results shown, the concentration of miR-125a-5p in EVs^miR^ was 148.27 copies/μL (Figure 2J-K), significantly higher than that of native EVs (3.55 copies/μL). Given that all other processes used to generate EVs were identical, we hypothesized that electroporating miR-125a-5p into EVs might result in these variations.

### EVs with miR-125a-5p inhibit neuron injury by suppressing the expression of the TLR4/NF-κB signaling by directly targeting *IKBKG*

**Figure 3:**
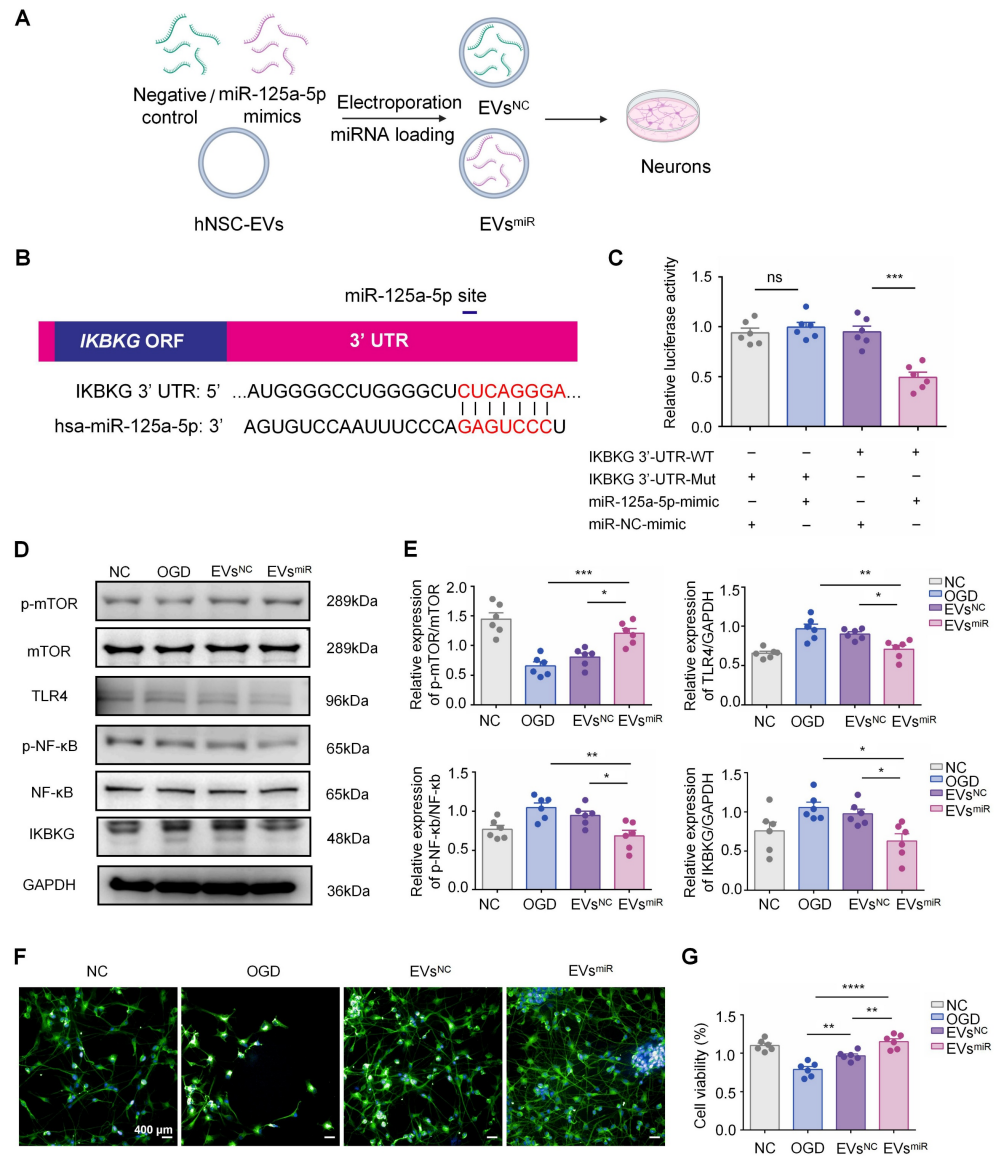
** Extracelluar vesicles (EVs) with miR-125a-5p suppressed the expression of TLR4/NF-κB signaling through directly targeting *IKBKG*.** (A) Schematic representations of the preparation of EVs^miR^ and EVs^NC^ for treating injured neurons. (B) Complementary sequences between miR-125a-5p and the 3′-UTR of *IKBKG* mRNA obtained using TargetScan. (C) Luciferase activity in the *IKBKG* 3′-UTR constructs and miR-125a-5p mimics (or a negative control miRNA mimic) cotransfected 293T cells (*n* = 6). (D-E) Western blotting of p-mTOR, mTOR, TLR4, NF-κb, p-NF-κb, and IKBKG expressions in neurons after EVs^miR^ treatment (*n* = 6). GAPDH expression levels were detected as an endogenous control. (F) Observation of neurites in neurons in NC, OGD, EVs^NC^, and EVs^miR^ groups using the Opera Phenix screening system. Scale bars, 400 μm. MAP-2 (green) and Hoechst 33342 (blue). (G) Cell viability of neurons analized using the CCK-8 assay (*n* = 6). All data are expressed as the mean ± SEM. *P < 0.05, **P < 0.01, ***P < 0.001, ****P < 0.0001; ns: not significant.

To further explore the mechanism underlying the miR-125a-5p of EVs involved in inflammation, miR-125a-5p mimics were loaded into EVs using electroporation (Figure 3A). To determine the direct targeting of miR-125a-5p to *IKBKG* mRNA, potential binding site in miR-125a-5p for the *IKBKG* 3ʹ-UTR were investigated using TargetScan (Figure 3B). Subsequently, a luciferase reporter assay was performed by cotransfecting cultured 293T cells with *IKBKG* 3ʹ-UTR constructs that contained the presumed binding site of miR-125a-5p, either with miR-125a-5p mimics or a negative control. The miR-125a-5p suppressed luciferase activity in the wild-type reporter construct but had no effect on the mutant 3ʹ-UTR (Figure 3C). These results suggest that miR-125a-5p directly targets to *IKBKG*, leading to the downregulation of its expression by binding to the specific site (CUCAGGGA) on the 3ʹ-UTR. The expression levels of IKBKG in neurons after OGD injury and EVs^miR^ treatment were detected using western blotting. The expression of IKBKG increased after OGD injury and was suppressed by miR-125a-5p-enriched EVs (EVs^miR^), suggesting that EVs^miR^ inhibited the expression of IKBKG in injured neurons through miR-125a-5p (Figure 3D-E). Additionally, we assessed neuronal proliferation using MAP-2 immunofluorescence staining and the CCK-8 assay. We found that treatment with EVs^NC^ and EVs^miR^ promoted the proliferation and repair of neurons injured by OGD. Following the treatment with EVs^miR^, a significant increase in MAP-2-positive cells was observed. Consistently, the CCK-8 assay demonstrated treatment with EVs^NC^ and EVs^miR^ significantly enhanced neuronal cell viability, with EVs^miR^ eliciting a notably stronger effect (Figure 3F-G). Thus, miR-125a-5p promotes neuronal proliferation by targeting *IKBKG*, resulting in efficient neural remodeling. We also performed qRT-PCR to assess the intracellular levels of miR-125a-5p following EVs^miR^ administration. The results confirm that treatment with EVs^miR^ markedly elevates the relative intracellular expression levels of miR-125a-5p via cellular uptake, allowing it to exert its subsequent effects (Figure S2).

To investigate the regulatory impact of miR-125a-5p on TLR4/NF-κB and mTOR signaling pathways by targeting *IKBKG*, we tested the effect of EVs^miR^ on neurons after OGD injury. Western blotting revealed that the levels of TLR4 and p-NF-κB were significantly downregulated, and the expression levels of p-mTOR were markedly upregulated using EVs^miR^ treatment (Figure 3D-E). Notably, no significant difference was observed between the OGD (injured neurons without treatment) and EVs^NC^ group. Additionally, there were no significant differences in the expressions of mTOR and NF-κB among the groups. Our study revealed that miR-125a-5p inhibited the phosphorylation of NF-κB and effectively decreased the activity of TLR4 signaling in neurons that sustained injury. These findings suggest that the activities of TLR4/NF-κB and mTOR signaling are mostly regulated using EVs with miR-125a-5p by directly targeting *IKBKG* to repair injured neurons.

### Increased miR-125a-5p in hNSC-EVs promote M2 polarization of microglia and mediate crosstalk between neurons and microglia *in vitro*

**Figure 4:**
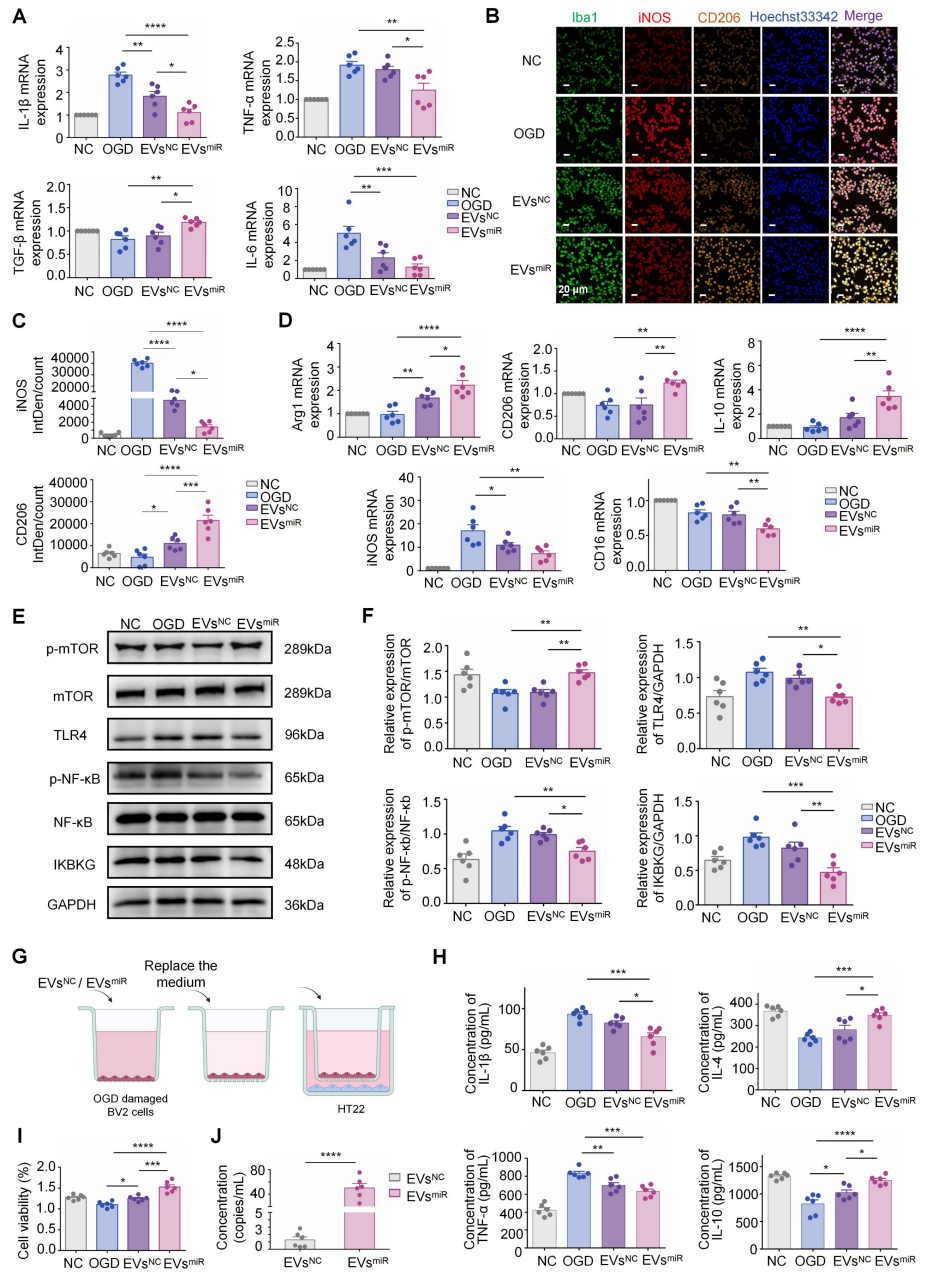
** MiR-125a-5p-enriched human neural stem cell extracellular vesicles (hNSC-EVs) promotes M2 polarization of microglia and mediates crosstalk between neurons and microglia *in vitro*.** (A) The mRNA expression levels of inflammation-related genes were detected using qRT-PCR in BV2 microglia in the negative control (NC), oxygen-glucose deprivation (OGD), EVs^NC^, and EVs^miR^ groups (*n* = 6). (B) Immunostaining of iNOS (red), CD206 (orange), and Iba1 (green) of BV2 cells of the four groups. Scale bars, 20 μm. (C) Fluorescence intensity ratio changes of CD206 versus iNOS of the four groups (*n* = 6). Images with a cell density higher than 30 cells per image were used for evaluation. (D) The mRNA expression levels of M1- and M2-related genes were detected using qRT-PCR in BV2 microglia in the different groups (*n* = 6). (E-F) Western blotting of p-mTOR, mTOR, TLR4, NF-κb, p-NF-κb, and IKBKG expressions in BV2 cells after EVs^miR^ treatment (*n* = 6). GAPDH expression levels were detected as an endogenous control. (G) Scheme of transwell study of co-culture of BV2 cells (upper chamber) and HT22 cells (lower chamber), created with BioRender.com. (H) Relative expression of pro-inflammatory factors (TNF-α and IL-1β) and anti-inflammatory factors (IL-4 and IL-10) analyzed using ELISA (*n* = 6). (I) Cell viability of HT22 cells (lower chamber) in a transwell study analyzed using CCK-8 assay (*n* = 6). (J) Concentration of miR-125a-5p in co-culture medium after EVs^miR^ treatment (*n* = 6 independent samples). The quantitation results are shown as the mean ± SEM of independent experiments. Analyzed using a one-way analysis of variance with Turkey′s multiple comparisons test, *P < 0.05, **P < 0.01, ***P < 0.001, ****P < 0.0001.

To investigate the effect of EVs with miR-125a-5p on CIR-induced neuroinflammation *in vitro*, an OGD model was established using BV2 cells. The injured BV2 cells were treated with EVs^miR^ or EVs^NC^. Treatment with EVs^miR^ significantly inhibited the inflammatory response by suppressing the expression of pro-inflammatory cytokines (TNF-α, IL-1β, and IL-6) and promoting the expression of anti-inflammatory cytokines (TGF-β) (Figure 4A), suggesting that increased miR-125a-5p in hNSC-EVs exerted the effect of inhibiting neuroinflammation. This may be related to the effect of EVs^miR^ on the modulation of microglia phenotype. As shown in Figure 4B and C, the EVs^miR^ groups showed a significant decrease in iNOS-positive microglia and higher fluorescence intensity of CD206 than those in the EVs^NC^ group, highlighting the impact of EVs^miR^ treatment on the M1/2 polarization of microglia/macrophages *in vitro*.

Additionally, we measured the expression levels of M1-related (*iNOS* and *CD16*) and M2-related (*Arg1, CD206*, and *IL-10*) genes in each group using RT-PCR. Compared with the EVs^NC^ group, the EVs^miR^ group showed a higher expression of M2 genes and lower expression of M1 genes (Figure 4D). Consequently, the findings revealed that EVs^miR^ could regulate the polarization of microglia/macrophages from the M1 to M2 phenotype and had a substantial impact on the ratio of anti-inflammatory to pro-inflammatory phenotypes in the OGD model. Taken together, these results demonstrated that hNSC-EVs shift the M1 phenotype to M2 in microglia by shuttling miR-125a-5p *in vitro*.

According to the GO and KEGG analysis results shown in Figure 1, the differentiated miRNAs of hypoxia-EVs and normoxia-EVs were significantly related to the regulation of TLR4 signaling pathways. To investigate whether the EVs miR-125a-5p inhibiting* IKBKG* gene expression can reduce activation of the TLR4-mediated signaling pathway, which is critical in the transition of macrophages and microglia to the anti-inflammatory M2 phenotype, we explored the possible underlying TLR4/NF-κB signaling pathways after administration of EVs^miR^ and EVs^NC^ in BV2 cells. Western blotting revealed that the levels of TLR4, p-NF-κB, and IKBKG were significantly downregulated, and p-mTOR expression was markedly upregulated with EVs^miR^ treatment (Figure 4E-F). MiR-125a-5p may be involved in hNSC-EV-mediated microglial polarization by targeting the mTOR/TLR4/NF-κB signaling cascade.

To validate the effects of microglia on recovery-associated phenotypes in neurons after miR-125a-5p in EVs treatment, a Transwell model with cocultured BV2 and HT22 cells was used to demonstrate the crosstalk between microglia and neurons (Figure 4G). OGD-induced BV2 cells in the upper chamber were treated with EVs^miR^ after reoxygenation and then cocultured with HT22 cells. Production of pro-inflammatory cytokines, including TNF-α and IL-1β, was significantly reduced (Figure 4H). However, the concentration of TNF-α in the EVs^miR^ group was slightly reduced compared with that in the EVs^NC^ group, but not significant. In addition, the levels of anti-inflammatory cytokines, including IL-4 and IL-10, increased after treatment, suggesting that EVs^miR^ reduced inflammation. These results demonstrate that hNSC-EVs could facilitate the transition of microglia from an ischemia-associated phenotype to a recovery-associated phenotype by shuttling miR-125a-5p. This transition may contribute to neuroprotection following ischemia reperfusion (Figure 4I). To further determine whether miR-125a-5p was directly delivered from microglia to neurons via EVs, we examined the abundance of miR-125a-5p in EVs isolated from the co-culture medium. The medium of BV2 cells treated with EVs^miR^ alone was replaced with fresh medium and co-cultured with HT22 cells. The co-cultured medium was collected and analyzed using ddPCR. The ddPCR results suggested that EVs miR-125a-5p was significantly increased in the EVs^miR^ administration group compared with that in the EVs^NC^ treatment group (Figure 4J). To further elucidate whether the crosstalk between neurons and BV2 cells was specifically induced by EVs^miR^, we performed qRT-PCR to measure the miR-125a-5p levels in co-cultured BV2 and HT22 cells. We observed a significant increase in miR-125a-5p expression in both cell types following EVs^miR^ treatment, whereas no statistically significant alterations in the relative expression levels of miR-125a-5p were detected in the other groups. Moreover, the EVs^inhibitor^ group demonstrated an obvious downregulation of relative miR-125a-5p expression levels, indicating effective suppression of miR-125a-5p activity (Figure S3A-B). These results indicated that miR-125a-5p is secreted by BV2 cells and delivered to neurons after EVs^miR^ treatment, consequently regulating neuronal repair.

### Increased miR-125a-5p in hNSC-EVs improves the neurological deficits in MCAO rats

**Figure 5:**
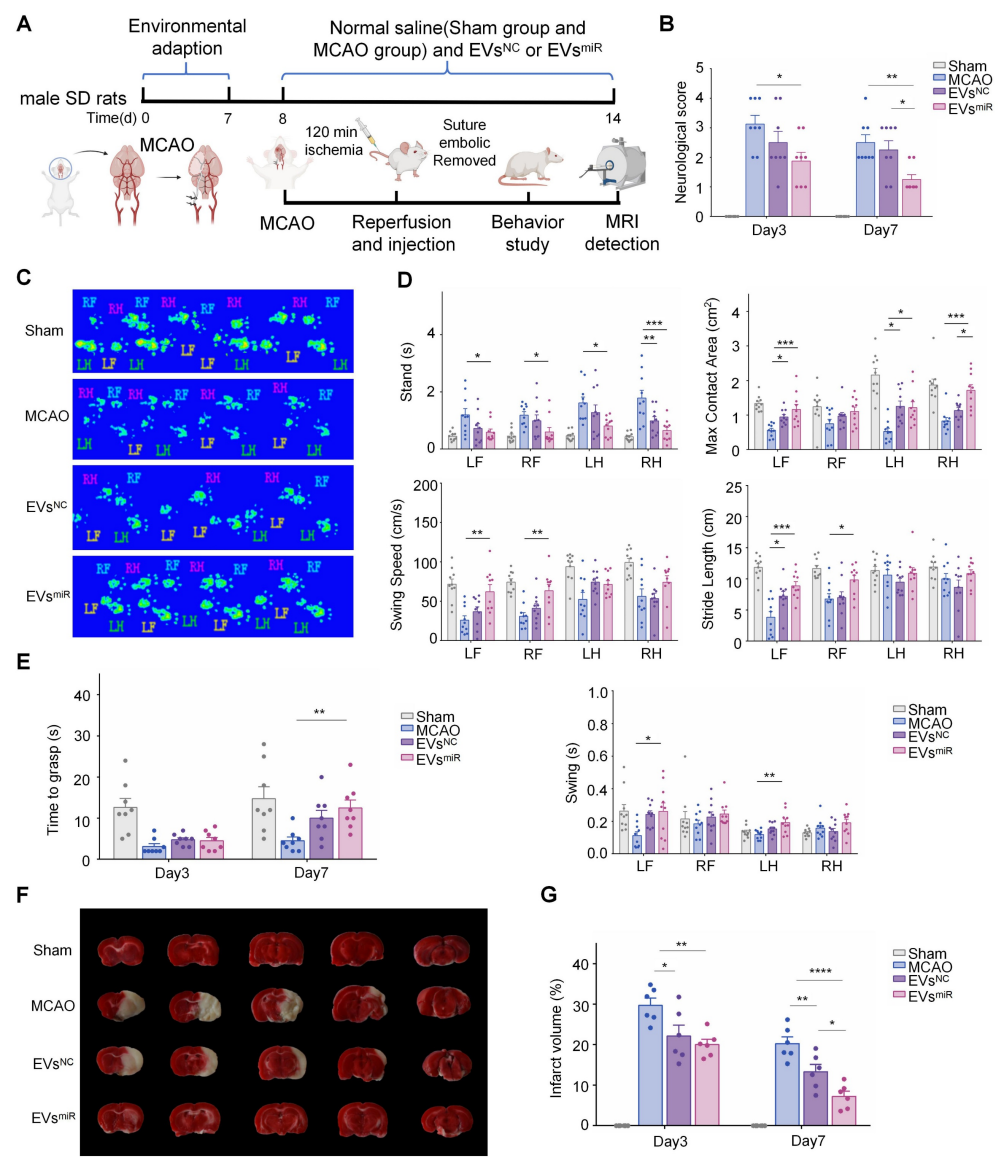
** Increased miR-125a-5p in human neural stem cell extracellular vesicles (hNSC-EVs) improves the neurological deficits in middle cerebral artery occlusion (MCAO) rats.** (A) Schematic timeline of the study of EVs^NC^ and EVs^miR^ treatment in cerebral ischemia reperfusion injury (CIRI) rats. Created with BioRender.com. *In vivo* brain reparation of the EVs^miR^ in MCAO-induced ischemia reperfusion injury. The sham group was taken as the control group, and rats in the other three groups were treated using MCAO and corresponding administration of saline, EVs^NC^, and EVs^miR^. (B) Quantification of neurological scores up to 3 and 7 days after CIRI (*n* = 8 animals per group). (C-D) Catwalk gait analysis was used to determine the locomotor recovery after EVs^NC^ and EVs^miR^ treatment. C, representative images of footprints after EVs^NC^ and EVs^miR^ treatment; D, quantitative analysis of the stance time, paw maximum contact areas, swing speed, stride length, and swing time. RF, right forelimb; RH, right hindlimbs; LF, left forelimb; LH, left hindlimbs. Print area, stride length, and swing speed represented the moving activity of rats in different experimental groups (*n* = 10 animals per group). (E) Quantification of the grasping capability test up to 3 and 7 days after CIRI grasping capability test (*n* = 8 animals per group). (F) Representative images of TTC staining of brain slices on day 7 after MCAO. (G) Quantitative results of cerebral infarct volume in MCAO rats with or without treatment at day 3 and 7 after MCAO (*n* = 6). All data are presented as means ± SEM. Analyzed using a one-way analysis of variance with Turkey's multiple comparisons test with *P < 0.05, **P < 0.01, ***P < 0.001, ****P < 0.0001.

A MCAO model was established to confirm the effect of EVs^miR^ on ischemia-reperfusion injury *in vivo* (Figure 5A). First, an In Vivo Imaging System was used to assess EVs biodistribution, which revealed that EVs successfully crossed the BBB and penetrated the brain parenchyma to exert their biological effects (Figure S4). We evaluated the functional recovery of rats treated with saline, EVs^NC^, or EVs^miR^ to explore whether miR-125a-5p-enriched EVs could have a positive impact on motor function following CIRI. Neurological deficit scores of the rats were determined using the Zea-Longa score. As shown in Figure 5B, the rats in the EVs^NC^ and EVs^miR^ groups demonstrated greater functional improvements than that of rats in the saline group. Additionally, the neurological impairment scores in the EVs^miR^ group were significantly lower than those in the EVs^NC^ group on day 7, indicating EVs^miR^ was an effective therapy in reducing acute injury.

To further study motor functional behavioral recovery, gait impairment in rats was evaluated at 3 and 7 days after injury. MCAO rats were unable to cross the platform normally 3 days after injury and significant alterations in the ratsʹ gait parameters were observed by day 7. Analysis of the digital gait assessment revealed that right MCAO led to noticeable impairments in the left limb, as indicated by the increased swing time along with decreased maximum contact area and brake time. In rats treated with EVs^miR^, coordinated movement of the forelimbs and hindlimbs was observed in Figure 5C. Compared with the MCAO controls, stride length considerably decreased in the forelimb during ischemia-reperfusion injury and recovered after 7 days of EVs^miR^ treatment. Additionally, EVs^miR^ treatment significantly enhanced multiple parameters of functional restoration, such as stand time, maximum contact area, swing duration, and average swing speed of the paws touching the glass plate, compared with those of the MCAO controls, especially for the left forelimb (Figure 5D). Finally, we used the grasping capability test to evaluate neurological recovery and found that the time taken by the forelimb to grasp the rope was increased in the EVs^miR^ group compared with that in the control group (Figure 5E). Thus, these findings demonstrated that EVs^miR^ significantly improved locomotor function loss due to MCAO. We assessed the infarct volume in MCAO rats using TTC staining, where viable brain tissue appeared red and infarcted regions remained white. Representative TTC staining images at 7 days post-MCAO are shown in Figure 5F. Quantitative analysis of the infarct volumes on days 3 and 7 revealed that the MCAO group exhibited significantly larger infarcts than that of the other groups. Treatment with EVs^NC^ and EVs^miR^ substantially reduced infarct size, with EVs^miR^ demonstrating the most pronounced therapeutic effect in attenuating infarct volume (Figure 5G). We also performed a biosafety evaluation of the treatment of rats. Immunohistological staining of major organs, including the heart, liver, spleen, lungs, and kidneys (Figure S5). Compared with the sham group, treatment with EVs^NC^ and EVs^miR^ caused little difference, indicating imperceptible damage to the organs. Taken together, our findings suggested that both EVs^NC^ and EVs^miR^ transplantation can promote functional behavioral recovery and reduce the infarct volume in rats after CIRI, with EVs^miR^ treatment showing a considerably more beneficial effect.

### Increased miR-125a-5p in hNSC-EVs inhibiting the TLR4/NF-κB signaling pathway by targeting *IKBKG* is confirmed *in vivo*

**Figure 6:**
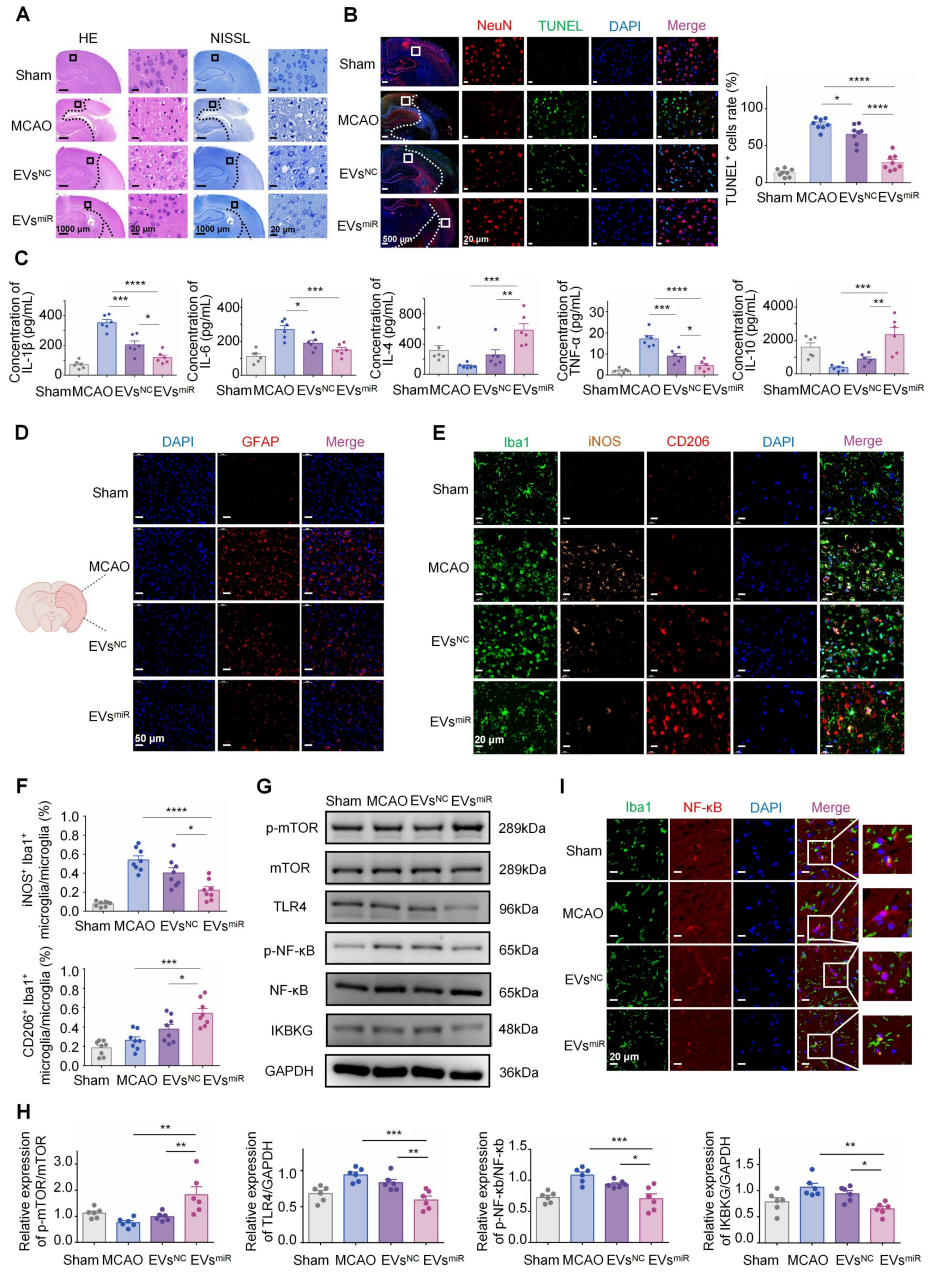
** Increased miR-125a-5p in human neural stem cell extracellular vesicles (hNSC-EVs) exerted a protective effect of inhibiting neuronal inflammation* in vivo.*** (A) Typical HE staining and NISSL staining photographs showed the degeneration and necrosis of the nerve cells in the peri-infarct cortex (CTX, black boxes) and destructive tissues indicated in the model group. Scale bars, 20 μm. (B) TUNEL, NeuN, and DAPI co-staining in four groups after corresponding treatment, and the number of TUNEL-positive cells was calculated (*n* = 8/group). TUNEL (green), NeuN (red), and DAPI (blue). Scale bars, 20 μm. Quantitative analysis was performed in the ischemic neurons in each group. (C) The concentrations of pro-inflammatory and anti-inflammatory cytokines, including IL-1β, IL-6, TNF-α, IL-4, and IL-10 in peri-infarct cortex of brain tissues in different groups were confirmed using ELISA (*n* = 6). (D) Glial scar evaluation by immunofluorescence GFAP staining of the ischemic penumbra for different groups. GFAP (red) and DAPI (blue). Scale bars, 50 μm. (E-F) Representative immunostaining image of Iba1 (green) and iNOS (orange)/CD206 (red) in the injured ischemic lesion areas at day 3 after injury and the analysis of iNOS/CD206-positive microglia/macrophage in the ischemic lesion area (*n* = 8/group). Scale bars, 20 μm. (G-H) Representative images and quantification of western blots for IKBKG, TLR4, and downstream NF-κB and mTOR signaling cascades in the peri-infarct cortex when administrating saline, EVs^NC^, and EVs^miR^ (*n* = 6). (I) Colocalization of NF-κB with microglia in different groups 3 days after modeling. Iba1 (green), NF-κB (red), and DAPI (blue). Scale bars, 20 μm. The data are presented as means ± SEM. Analyzed using a one-way analysis of variance with Turkey's multiple comparisons test with *P < 0.05, **P < 0.01, ***P < 0.001, ****P < 0.0001.

To investigate the involvement of miR-125a-5p in the progression of functional behavioral recovery and suppression of neuroinflammation after CIRI *in vivo*, several experiments were performed. The results of H&E and NISSL staining (Figure 6A) indicated a notable reduction in the density of nerve cells in the peri-infarct cortex in the MCAO group compared with that in the control group. Conversely, the EVs^miR^ group exhibited a considerably higher number of nerve cells than that in the MCAO group. In addition, neuronal mortality at the infarct boundary was investigated using TUNEL staining and NeuN, a marker specifically for neurons. Neurons in both therapy groups displayed less TUNEL staining than those in the MCAO group. In accordance with the occurrence of brain infarction, the EVs^miR^ group exhibited a significant reduction in TUNEL^+^ and TUNEL^+^ NeuN^+^ cells compared with those in the MCAO group (Figure 6B). Quantitative analysis of brain tissue with TUNEL/DAPI co-staining revealed that EVs^miR^ exhibited a significant inhibitory effect on necrosis and apoptosis of brain cells within infarcted regions. These results indicate that EVs^miR^ mitigates CIR-induced brain injury caused by CIRI. We also used qRT-PCR to quantify its expression levels within the brain tissue following EVs^miR^ administration, thereby demonstrating that miR-125a-5p successfully penetrated the brain tissue to mediate its downstream effects (Figure S6).

The effect of elevated miR-125a-5p levels in EVs on neuroinflammation was assessed by measuring inflammatory mediator expression levels in the injured hemisphere of MCAO rats. MCAO induced neuroinflammation in the affected brain, marked by an upregulation of pro-inflammatory cytokines (TNF-α, IL-1β, and IL-6) and a reduction in anti-inflammatory cytokines (IL-4, IL-10). The data indicated that the administration of both EVs^NC^ and EVs^miR^ significantly reduced the levels of pro-inflammatory cytokines and increased the levels of anti-inflammatory cytokines compared with those in the saline group. Nevertheless, EVs^miR^ treatment further enhanced the release of anti-inflammatory cytokines and suppressed the release of pro-inflammatory cytokines more effectively than those in the EVs^NC^ group (Figure 6C).

During ischemia-reperfusion injury, there is rapid accumulation of activated glial cells in the brain, leading to glial scar formation and disruption of the self-repair capabilities of brain tissues. The expression of GFAP, a biomarker associated with astrocytes, was assessed in the ischemic penumbra of MCAO rats following different therapies (Figure 6D). In the penumbra sections of the MCAO rats, GFAP expression was substantially elevated compared with that in the sham-operated group, suggesting an increase in the number of GFAP-positive cells. However, administration of EVs^miR^ effectively mitigated this effect.

As shown in Figure 6E and F, there was a noticeable reduction in iNOS-positive microglia and an elevated level of CD206 in the microglia/macrophages in the lesion areas on day 3 after injury in the EVs^NC^ and EVs^miR^ groups compared with those in the MCAO group.

Notably, the EVs^miR^ group showed a tendency towards a lower ratio of iNOS-positive microglia/macrophages and higher ratio of CD206-positive microglia/macrophages than those in the EVs^NC^ group. This finding underscored the impact of EVs^miR^ treatment on the M1/2 polarization of microglia/macrophages *in vivo*. Therefore, our results indicated that EVs^miR^ had a notable impact on the proportion of anti-inflammatory to pro-inflammatory characteristics following CIR and could alter the polarization of microglial/macrophages from the M1 to M2 phenotype.

To verify whether miR-125a-5p inhibits the TLR4/NF-κB signaling pathway by targeting *IKBKG in vivo*, it influences microglial polarization and modulates neuroinflammatory responses. Consequently, we investigated the potential effect of miR-125a-5p on the TLR4/NF-κB signaling pathways following the administration of EVs^NC^ and EVs^miR^ in the ischemia tissues of rats after CIRI. As shown in Figure 6G and H, TLR4 and p-NF-κB protein levels were significantly increased after MCAO treatment, while EVs^miR^ significantly inhibited the expression of TLR4, p-NF-κB, and IKBKG and promoted expression of p-mTOR compared with those in the MCAO group. Our results indicated that EVs played a role in neuroinflammation and microglia polarization through the delivery of miR-125a-5p, which targeted *IKBKG* via the TLR4/NF-κB and mTOR signaling cascade. This result was consistent with the verified pathway of miR-125a-5p *in vitro*.

Finally, to further confirm the mechanism of EVs regulation by miR-125a-5p on microglia polarization and neuroinflammation, we conducted double fluorescence staining in the ischemic penumbra using Iba1 and NF-κB, to evaluate NF-κB activation through its spatial localization. In the MCAO and EVs^NC^ groups, NF-κB was predominantly localized within the nucleus, reflecting its active state, with limited cytoplasmic presence. Treatment with EVs^miR^ significantly inhibited this nuclear translocation, maintaining NF-κB localization in the cytoplasm, thereby suppressing its activation (Figure 6I). Our findings provided additional evidence that EVs inhibited the NF-κB signaling pathway via miR-125a-5p, which impacted the entry of NF-κB into the nucleus. Therefore, it plays a key role in microglial polarization and neuroinflammation.

### Increased miR-125a-5p in hNSC-EVs repairs injured neural microstructures

**Figure 7:**
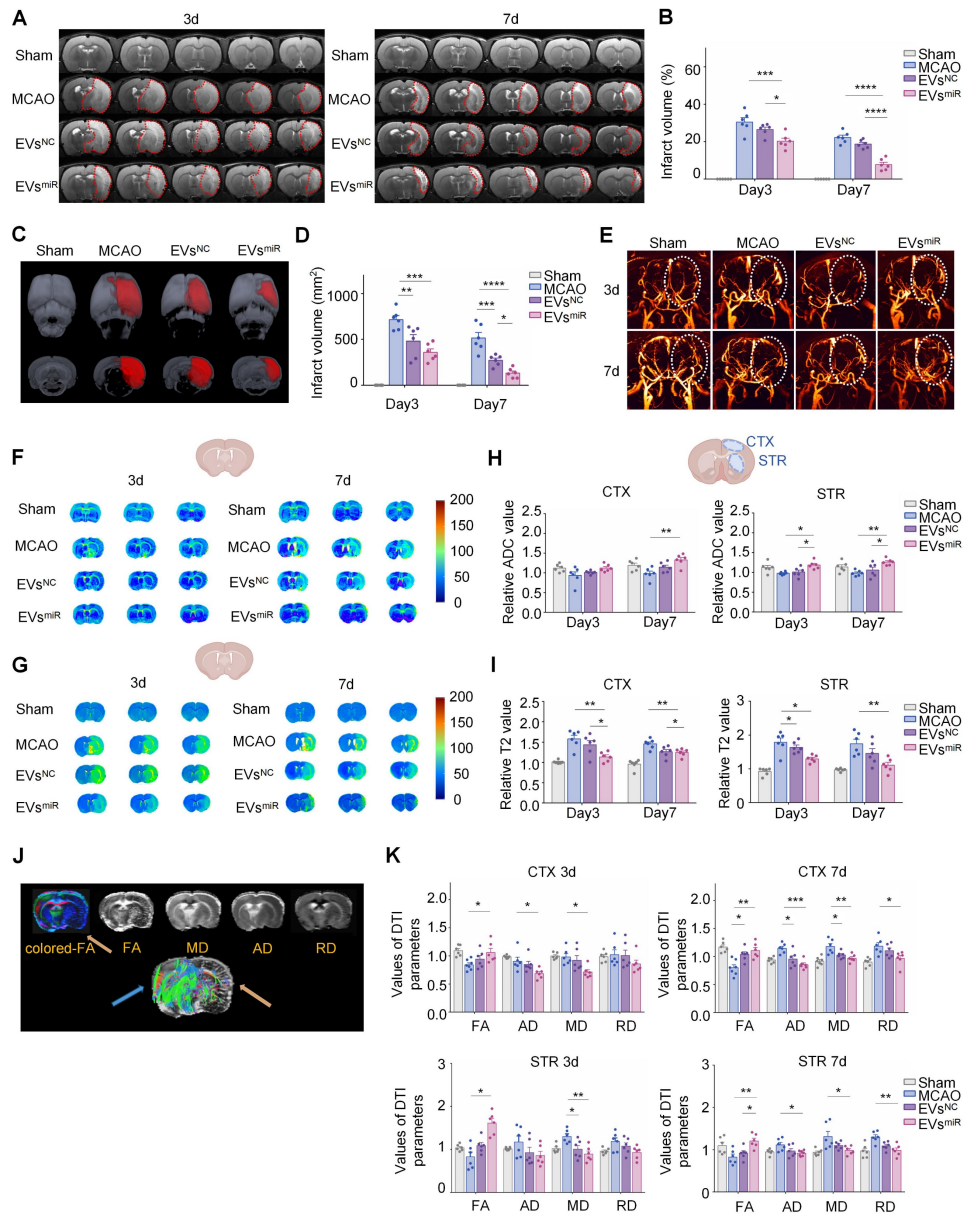
**Magnetic resonsance imaging (MRI) monitoring of each treatment on middle cerebral artery occlusion (MCAO) rats with 2-h ischemia.** (A) Representative T2-weighted imaging (T2WI) of the brains in sham-operated and MCAO rats with different treatments on days 3 and 7. Red curves represent the infarct regions. (B) The brain infarct volume in each treatment group on days 3 and 7 (*n* = 6). (C-D) Three-dimensional lesion volumes were computed from T2 maps on days 3 and 7. Ischemic (red) tissues were automatically identified (*n* = 6). (E) Representative vascular MRI of the cerebral vessels in sham-operated rats and MCAO rats with different treatments to characterize the microcirculatory perfusion on days 3 and 7. White curves show the ischemic lesions. (F-G) Representative apparent diffusion coefficient (ADC) mapping and T2-mapping images of the brains from each treatment group at predetermined time points. (H) Selected regions of interest (ROIs) for measuring the signal intensity of MCAO/R in the brains. ROIs for cortex and striatum were symmetrically selected in the left and right hemispheres to compare the contrast-to-noise ratio. Quantitative comparison of ADC values from each treatment group (*n* = 6). (I) Selected ROIs for measuring the signal intensity of MCAO/R in the brain. ROIs for cortex and striatum were symmetrically selected in the left and right hemispheres to compare the contrast-to-noise ratio. Quantitative comparison of T2 values from each treatment group (*n* = 6). (J) Typical colored fractional anisotropy (FA), axial diffusivity (AD), radial diffusivity (RD), and mean diffusivity (MD) maps were obtained in the cortex (CTX) and striatum (STR). Representative images of three-dimensional fiber tracking were performed using the Trackvis and Diffusion Toolkit software. (K) Quantitative analysis of the relative FA, AD, MD, and RD in the CTX and STR (*n* = 6). All data are presented as means ± SEM. Analyzed using a one-way analysis of variance with Turkey's multiple comparisons test, *P < 0.05, **P < 0.01, ***P < 0.001, ****P < 0.0001.

MRI was used to evaluate the therapeutic effects of EVs^miR^ in MCAO rats. T2W images revealed no apparent infarctions (hypersignals) in sham-operated rats on days 3 and 7, whereas clear ischemic regions were visible in the MCAO group. Compared with the MCAO group, MCAO rats treated with EVs^NC^ and EVs^miR^ exhibited varying degrees of reduction in cerebral infarction, with the EVs^miR^ group showing the most significant decrease. Notably, the infarcts of the EVs^miR^ group decreased on day 7 and remained considerably lower than those of the other groups (Figure 7A-B).

The total ischemic lesion volumes calculated from T2 maps on day 3 are shown in Figure 7C. T2WI showed T2 hyperintensities in the ischemic area of the MCAO rats, indicative of edema and extensive lesions. In contrast, all treatments, particularly EVs^NC^ and EVs^miR^, reduced lesion volumes that were apparent only in a small portion of the cortex and striatum. Compared with the MCAO group, EVs^NC^ and EVs^miR^ treatments dramatically decreased the three-dimensional lesion volumes (Figure 7C). The total lesion volumes were considerably reduced by all treatments compared with those in the MCAO group, and the lowest volume was observed in the EVs^miR^ group, as depicted in Figure 7D. These results indicated that EVs^miR^ effectively reduced the volume of infarct regions and decelerated their progression.

The 2D-TOF-MRA was performed to assess microvascular reperfusion in the ischemic hemisphere. The sham group exhibited normal microvascular perfusion in the right brain, whereas the MCAO group showed significant blockage and minimal blood circulation. The administration of EVs^NC^ and EVs^miR^ resulted in a notable enhancement of microvascular reperfusion in MCAO rats. In particular, the EVs^miR^ group exhibited a microvascular reperfusion improvement comparable to that in the sham group (Figure 7E). Taken together, these findings highlighted the significant efficacy of EVs^miR^ in alleviating the “no-reflow” of microvasculature caused by reperfusion injury.

Cerebral infarction limits the mobility of water molecules, leading to a decrease in the ADC value and an increase in T2 value in ischemic brain tissue 34. The ADC and T2 values of each group were subsequently determined using DWI (Figure 7F) and T2-mapping imaging (Figure 7G), respectively. Compared with the MCAO group, the EVs^miR^ group exhibited an increase in ADC values, although the changes were not statistically significant on day 3 in the cortex. However, there was a significant increase in the ADC values in the cortical and striatal regions on day 7. The T2 values of the EVs^miR^ group were significantly decreased. Nevertheless, in the other groups, the alterations in ADC and T2 values were minimal (Figure 7H-I), providing additional evidence for the superior therapeutic benefits of EVs^miR^ in ischemia-reperfusion injury.

DTI was used to assess the alterations in the microstructure of the axons (Figure 7J). Initially, FA determined using DTI, was used to characterize changes in the microstructure of axons. Quantitative analysis revealed a significant decrease in the relative FA (rFA) in the perilesional cortex and striatum of MCAO rats compared with those in the sham group, but this was notably reversed by EVs^miR^ treatment (Figure 7K). Relative AD (rAD) and relative RD (rRD) were used to examine changes in axons and myelin sheaths, respectively. The DTI results indicated increased rAD and rRD in the peri-infarct cortex and striatum of the model rats compared with those in the sham rats. Administration of EVs^miR^ considerably lowered rAD in the peri-infarct cortex relative to that in the model rats and similarly reduced rAD in the striatum on day 7 (Figure 7K). Additionally, EVs^miR^ notably reduced rRD in both the peri-infarct cortex and striatum compared with that in the model group on day 7. Comapred with EVs^miR^ to the model group, the relative MD (rMD) was significantly lower in the peri-infarct cortex and striatum. These findings indicate that EVs^miR^ has the potential to mitigate axonal microstructural injury in rats with ischemia.

## Discussion

CIRI leads to neuronal cell death and the release of injury-related molecules, triggering intense localized inflammation in the injured brain region and resulting in severe neurological deficits 3. Due to the complex pathophysiological characteristics of CIRI, there are no effective treatments for serious maladies in clinical practice. Our preliminary study showed that hNSC-EVs have therapeutic effects in the treatment of CIRI 16, but the underlying mechanism remains unknown. The present study revealed that miRNA-125a-5p might be a key molecule in EVs for the treatment of CIRI in the MCAO model, and miRNA-125a-5p-enriched EVs (EVs^miR^) could attenuate the inflammatory response of injured microglial cells and promote the polarization of microglial cells from M1 to M2 through the delivery of miRNA-125a-5p. We also revealed that EVs^miR^ attenuate the inflammatory response and protect the ultrastructure of neuronal axons by maintaining neuron-microglial cell interactions during brain homeostasis. In addition, we innovatively found that miRNA-125a-5p inhibited mTOR and TLR4/NF-κB pathways by regulating *IKBKG*, thereby suppressing neuroinflammation, attenuating neuronal injury and restoring neurological function in MCAO rats. Thus, the combination of miRNA-125a-5p and hNSC-derived EVs may be a promising strategy for treating CIRI. The molecular mechanisms of the inflammatory response after CIRI revealed in the present study would provide clinical guidance for early intervention after cerebral ischemia.

EVs may be involved in the pathogenesis and treatment of stroke by delivering miRNAs, proteins, and other components 35-37. Several studies have demonstrated that miRNAs are one of the main functional components of EVs, and may play crucial roles in cell communication and the regulation of biological functions 38. A previous study reported a significant difference in the expression of miR-125a-5p in the blood of intracerebral hemorrhage and ischemic stroke rats, suggesting that miR-125a-5p is a common biomarker of stroke 39. Circulating endothelial microvesicles (EMVs) and EMV-miR-125a-5p were found to be strongly associated with ischemic stroke onset, progression, subtypes, and severity, and may serve as new biomarkers and therapeutic targets for ischemic stroke, especially when used in combination 40. However, the mechanisms underlying CIRI regulation by miR-125a-5p in EVs have not yet been explored. Using high-throughput sequencing analysis, we found for the first time that miR-125a-5p, which is upregulated in EVs from hypoxic hNSCs, is a modulator of neuroinflammation and injured neuron repair.

Excessive inflammatory responses can increase neuronal apoptosis and aggravate neurological deficits; TLRs play important roles in inflammatory responses 41. However, the precise role of TLRs remains to be elucidated because of their complex mechanisms in mediating neuronal injury. Activation of the TLR4/NF-κB signaling pathway is crucial for inflammatory immune responses. Here, we demonstrated that EVs^miR^ could inhibit post-ischemic inflammatory responses and thus reduce ischemia-induced neuronal injury by downregulating the TLR4/NF-κB signaling pathway. mTOR is a serine/threonine kinase involved in cell migration, protein synthesis, proliferation, and autophagy of cells 42. A previous study showed that morphine-induced neuroprotection in hippocampal neurons was accomplished by mTOR phosphorylation, which consequently decreased apoptosis 43. After EVs^miR^ treatment in MCAO rats, increased numbers of NeuN^+^ cells were found in the peri-infarct cortex, which rescued ischemic tissue neuronal cells. Overall, the MRI findings, along with the histological results, strongly supported that EVs^miR^ benefited vascular remodeling, improved collateral flow, and repaired the microstructure of axons. These results provide further understanding for the biological functions of miR-125a-5p during neuronal repair. IKBKG is the regulatory subunit of the IKK inhibitor complex that is essential for activating the NF-κB pathway involved in inflammation, immunity, and cell survival 44. Mutations in this gene cause several immunodeficient types of diseases, such as incontinentia pigmenti, hypertrichosis, and ectodermal dysplasia 45. In previous studies, *IKBKG*, a gene that may be involved in caspase-related apoptosis, was specifically regulated in female patients 5 h after a stroke 46. However, the role of *IKBKG* in CIRI has not been reported. Although hNSC-EVs inherently carry a diverse array of endogenous biomolecules, they can intrinsically modulate key signaling pathways, such as mTOR, TLR4, NF-κB, and IKBKG, to a limited extent. This baseline bioactivity accounts for the modulatory effects observed in the EVs^NC^ group in our study (Figures 3 and 4), which is consistent with references 47 demonstrating that native EV cargo profoundly influences recipient cell signaling and function. However, the therapeutic effects of EVs loaded with miR-125a-5p were markedly greater than those of EVs^NC^. We found that miR-125a-5p significantly suppresses ischemia-induced inflammation and neuronal death by targeting *IKBKG*. Taken together, these data indicated that the overexpression of miR-125a-5p played an important regulatory role in neuroprotection. Our results revealed that *IKBKG* was a direct and functional target gene of miRNA-125a-5p. EVs regulate IKBKG through miR-125a-5p to inhibit the TLR4/NF-κB signaling pathway and exert neuroprotective effects against cerebral ischemia, emphasizing the critical role of miR-125a-5p in neuroinflammation and neuronal death after CIRI.

Inflammatory response is an inevitable stage in the pathophysiological progression of CIRI. MiR-125a-5p inhibited the recruitment of macrophages to the inflamed retina by regulating Ninj1, protecting the vascular integrity of macrophages and thus attenuating inflammatory diseases and diabetic retinopathy 48. We found that EVs^miR^ effectively reduced the release of inflammatory factors, promoted the release of anti-inflammatory factors, and alleviated the neuroinflammatory responses after CIR. The rapid transition of microglia from M1 to M2 or the promotion of M2 polarization of quiescent microglia after the initiation of the healing process can inhibit excessive inflammatory responses in the brain and improve neurological deficits 9, 49. MiR-125a-5p was highly expressed in M2c macrophages compared with that in M0 and M1 macrophages in an *in vitro* model of human monocyte-macrophage differentiation 50. Although previous studies have linked mutations or dysregulation of miR-125a-5p to various disorders, its specific functions in the CNS, especially in the modulation of microglial polarization following CIR, have not been elucidated. Taken together, our findings indicate that EVs enriched with miR-125a-5p promote a microglial shift from the M1 to M2 phenotype, and improve neurological recovery following CIRI, and EVs act as biological vectors for the delivery of biologically functional miR-125a-5p into recipient microglia. These results showed that miR-125a-5p plays an important role in the regulation of microglial polarization, which is consistent with our finding that miR-125a-5p positively regulates the biological functions of macrophages.

Microglia-neuron communication is essential for homeostasis in the developing and adult CNS. In cerebral ischemic injury, microglia-neuron communication is most likely significant in both the acute phase, which involves altered neuroinflammation and cell death, and chronic phase, which includes the rerouting of neuronal circuits 51. According to emerging researches, microglia play an important role in neural homeostasis. Meanwhile, neuronal activity has been demonstrated to trigger microglial action via “on” and “off” signals. Microglia and neurons exchange numerous chemicals, such as intracellular signaling molecules, through reciprocal release of EVs 52. After hypoperfusion, miR-125a-5p supplied by EVs may act as a “help me” signal to microglia in the hypoxic environment. In the present study, we discovered that after OGD treatment, the release of miR-125a-5p in EVs was upregulated in primary hNSCs and adding miR-125a-5p-enriched EVs to microglial cultures could partly shift the expression of M2-microglial markers. We also found that ischemic hNSCs could contribute to M2-microglial polarization after MCAO by delivering miR-125a-5p of EVs, and inhibiting TLR4 and NF-κB signaling pathways. Previous studies have suggested that EVs produced from M2-microglial cells may have positive benefits. MiR-125a-5p was substantially expressed in M2c macrophages 50. Our findings demonstrated that miR-125a-5p expression was markedly increased in BV2 cells when EVs^miR^ was administered. Then, the activated microglia transmitted miR-125a-5p from EVs to neurons, consequently limited secondary neuronal injury caused by apoptotic or stressed neurons through mTOR/TLR4 and NF-κB signaling pathways and its downstream target, IKBKG. Taken together, based on the elevated EV miR-125a-5p levels in hypoxic hNSCs, we found that a systemic feedback regulatory mechanism may be involved in CIR, which, through miR-125a-5p delivery, leads to compensatory protection against neuronal injury.

Despite these promising findings, the present study has several limitations. Future studies should include competitive inhibition experiments to further validate the specificity of miRNA functions. Additionally, we plan to investigate the effects of miRNA treatment on behavioral recovery during the chronic phase following CIRI to better understand its long-term therapeutic potential. Building on our current results, future studies could focus on engineering EVs for the optimized delivery of EVs^miR^. Compared with nanoparticle-based miRNA delivery systems, EVs^miR^ demonstrates superior biocompatibility, enhanced cellular uptake, and improved ability to cross biological barriers, enabling more effective and targeted miRNA delivery 53, 54. These advantages suggest that engineered EVs are a promising platform for advancing miRNA-based therapies with improved efficacy and safety.

## Conclusion

We discovered and described a novel regulatory network directed by EV-delivered miR-125a-5p that regulates and synchronizes microglial polarization, neuronal repair, and remodeling upon inflammatory stimulation. Our work not only identified the cell-type spanning effect of miRNA shuttling between cell types via EVs of hNSCs but also revealed that miR-125a-5p, although differently regulated in neurons and microglia, guides biological processes in a synergistic and mutually reinforcing manner. Our study provides valuable insights into hNSC-EV miRNAs and their potential applications in treating CIRI.

## Supplementary Material

Supplementary figures.

## Abbreviations

CIRI: cerebral ischemia-reperfusion injury
EVs: extracellular vesicles
hNSCs: human neural stem cells
BBB: blood-brain barrier
CNS: central nervous system
EVs^miR^: miR-125a-5p enriched EVs
EVs^NC^: negative control enriched EVs
MCAO: middle cerebral artery occlusion
NTA: nanoparticle tracking analysis
TEM: transmission electron microscopy
OGD: oxygen-glucose deprivation
MRI: magnetic resonance imaging
TTC: 2,3,5-triphenyltetrazolium chloride
TUNEL: terminal-deoxynucleoitidyl transferase dUTP nick end labeling
TLR4: Toll-like receptor 4
CTX: cortex
STR: striatum
