# Supplementary material for: MiR-125a-5p in extracellular vesicles of neural stem cells acts as a crosstalk signal modulating neuroinflammatory microenvironment to alleviate cerebral ischemia-reperfusion injury
Source: Theranostics. 2025 Jun 12;15(14):7064–89. doi: 10.7150/thno.115993 (PMC12203920; doi:10.7150/thno.115993)
Supplement: Supplementary file 1 — Supplementary figures. [file thnov15p7064s1.pdf]

Supplementary Information

MiR-125a-5p in extracellular vesicles of neural stem cells acts as a crosstalk signal modulating neuroinflammatory microenvironment to alleviate cerebral ischemia-reperfusion injury

Qingyue Liu[a], Heran Ma[b], Jing Liao[a], Zihan Zhu[a], Hongyuan Chen[c], Dong Sun[d], Longkun Wang[e], Lu Lu[f], Xiaowei Chen[g], Xinke Zhang[a,]*, Fengshan Wang[a,]*

[a]Key Laboratory of Chemical Biology (Ministry of Education), Institute of Biochemical and Biotechnological Drug, School of Pharmaceutical Sciences, Shandong University, Jinan 250012, China.

[b]Qilu Cell Therapy Technology Co., Ltd, Jinan 250000, China.

[c]Department of General Surgery, Shandong Provincial Hospital Affilated to Shandong First Medical University, Jinan 250021, China.

[d]Department of Gastrointestinal Surgery, Shandong Cancer Hospital and Institute, Shandong First Medical University and Shandong Academy of Medical Science, Jinan 250012, China.

[e]Department of Pharmacy, Tianjin Anding Hospital, Tianjin 300000, China.

[f]Department of Pharmacy, Peking Union Medical College Hospital, Beijing 100010, China.

[g]Beijing Yinfeng Dingcheng Biological Engineering Technology Limited Liability Company, Beijing 100176, China.

*Corresponding author. Tel: +86 531 88382658; fax: +86 531 88382548; E-mail addresses: zhangxinke@sdu.edu.cn (X. Zhang); fswang@sdu.edu.cn (F. Wang).

SUPPLEMENTAL FIGURES

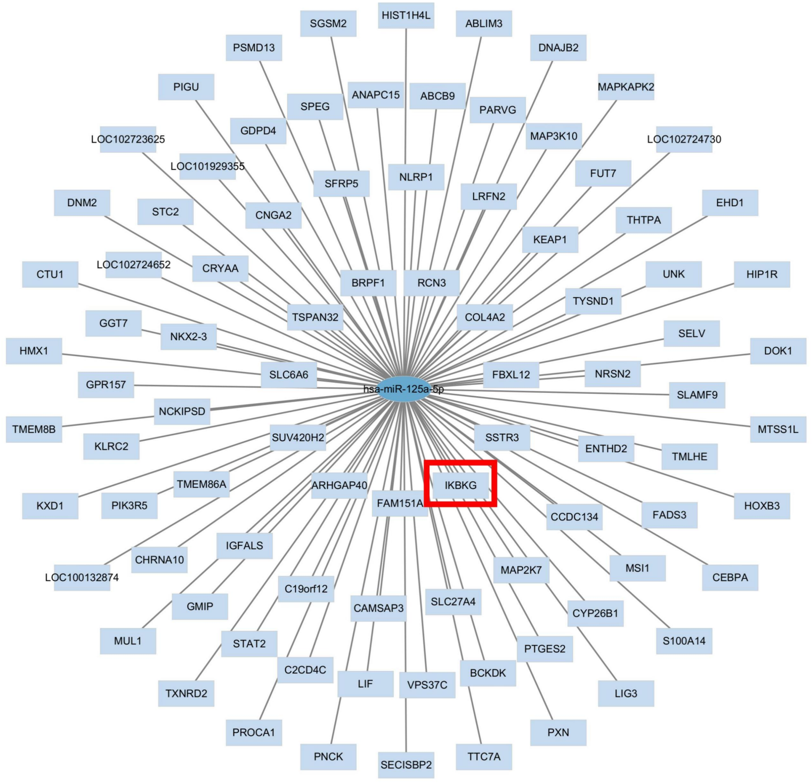

Figure S1 MiRNA-gene network was performed between hNSC-derived EVs hsa-miR-125a-5p and its key targeted genes by Cytoscape.

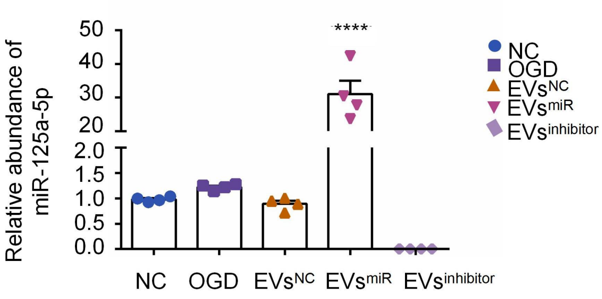

Figure S2 Comparison of the relative contents of miR-125a-5p in the neurons after different treatment post OGD injury by qRT-PCR (*n* = 4).

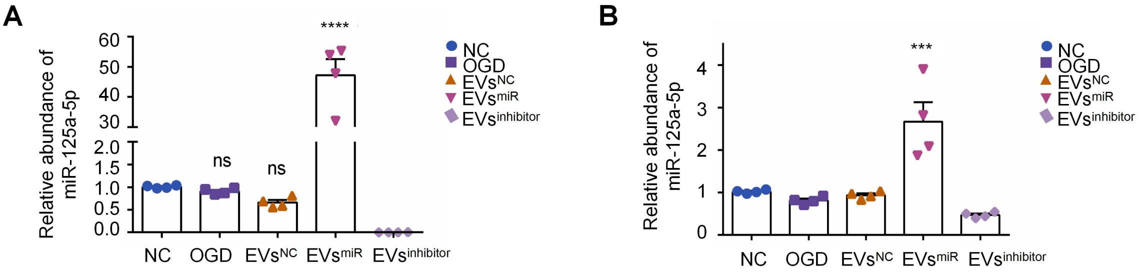

Figure S3 Comparison of the relative contents of miR-125a-5p in the co-culture model of (A) BV2 cells (upper chamber) and (B) HT22 cells (lower chamber) after different treatment post OGD injury by qRT-PCR (*n* = 4).

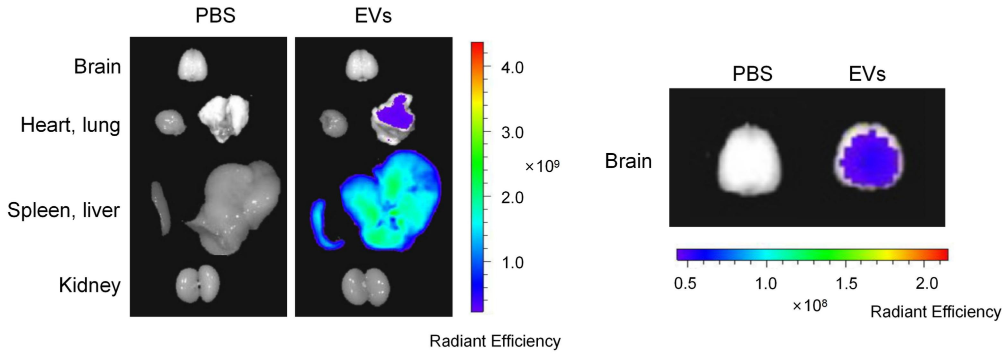

Figure S4 Biodistribution of EVs in major organs 24 h post intravenous administration in rats.

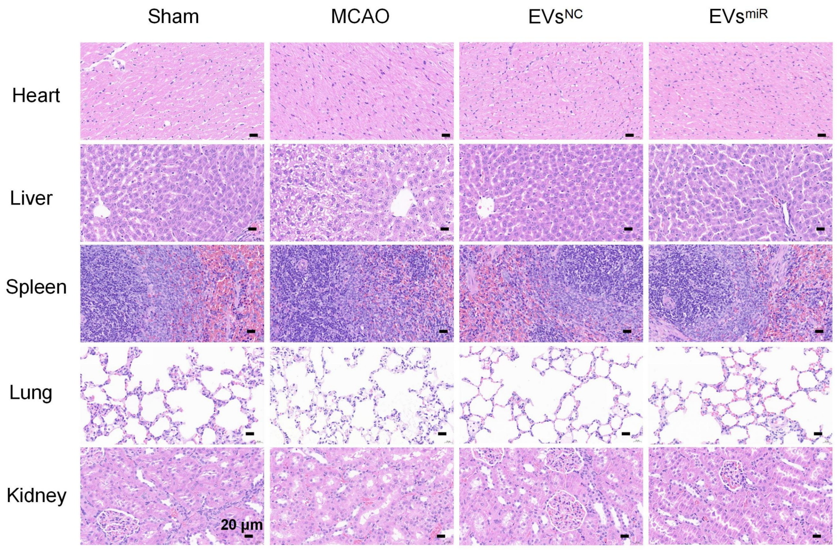

Figure S5 Hematoxylin and eosin (H&E) staining of heart, kidney, liver, lung and spleen tissue sections from sham group, saline treated MCAO group, and MCAO rats treated with EVs^{NC} and EVs^{miR}. Scale bar: 20 μm.

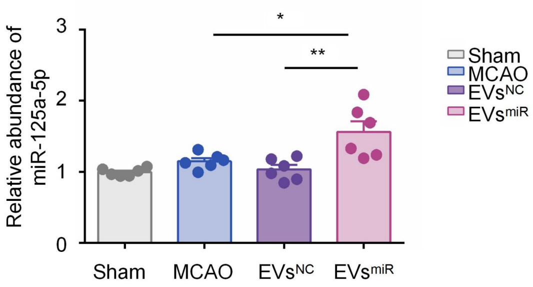

Figure S6 Comparison of the relative contents of miR-125a-5p in the MCAO rats after different treatment (*n* = 6).
